# Inferring functional units in ion channel pores via relative entropy

**DOI:** 10.1007/s00249-020-01480-7

**Published:** 2021-02-01

**Authors:** Michael Schmidt, Indra Schroeder, Daniel Bauer, Gerhard Thiel, Kay Hamacher

**Affiliations:** 1 Department of Physics, TU Darmstadt, Karolinenpl. 5, 64289 Darmstadt, Germany; 2 Department of Biology, TU Darmstadt, Schnittspahnstr. 10, 64287 Darmstadt, Germany; 3 Department of Physics, Department of Biology, Department of Computer Science, TU Darmstadt, Schnittspahnstr. 10, 64287 Darmstadt, Germany

**Keywords:** Potassium channel (pores), Mechanical coupling relative entropy, Anisotropic network model

## Abstract

Coarse-grained protein models approximate the first-principle physical potentials. Among those modeling approaches, the relative entropy framework yields promising and physically sound results, in which a mapping from the target protein structure and dynamics to a model is defined and subsequently adjusted by an entropy minimization of the model parameters. Minimization of the relative entropy is equivalent to maximization of the likelihood of reproduction of (configurational ensemble) observations by the model. In this study, we extend the relative entropy minimization procedure beyond parameter fitting by a second optimization level, which identifies the optimal mapping to a (dimension-reduced) topology. We consider anisotropic network models of a diverse set of ion channels and assess our findings by comparison to experimental results.

**Electronic supplementary material:**

The online version of this article (10.1007/s00249-020-01480-7) contains supplementary material, which is available to authorized users.

## Introduction

Coarse-grained models are established tools in a variety of applications in structural biophysics (Tozzini 2005; Bahar and Rader 2005; Shell 2008; Schlick 2010; Saunders and Voth 2013; Noid 2013; Kmiecik et al. 2016; Shell 2016). Their simplified structure allows investigation of important functional, dynamical, and structural properties of biomolecules including proteins, which are too large for finer grained approaches like molecular dynamics (MD) simulations. In coarse-grained schemes, less important modes and residues are eliminated from the model, so that the analysis can be focused on the larger scale dynamics of a protein.

Coarse-grained models were frequently used in the past to uncover dynamic structure/function correlations in ion channel proteins (Shi et al. 2006; Hamacher and McCammon 2006; Hamacher 2008; Bahar 2010; Stansfeld and Sansom 2011; Dryga et al. 2012; Das et al. 2014). Previous work (Shen et al. 2002; Shrivastava and Bahar 2006; Hoffgaard et al. 2015; Weißgraeber et al. 2016; Gross et al. 2018) also analyzed anisotropic network models (ANMs) of model $\hbox {K}^+$ channel pores. Shen and co-workers (Shen et al. 2002) used the model $\hbox {K}^+$ channel KcsA to uncover the main conformational motions in the channel for gating. By normal-mode analysis of this channel, they discovered two pivot points in the inner transmembrane domain, which undergo conformational changes during channel gating. In a follow-up study, the pores of different $\hbox {K}^+$ channels were analyzed again for common conformational changes, which underlie gating (Shrivastava and Bahar 2006). Normal-mode analysis of a Gaussian network model of the channel pores revealed a common pattern of intrinsic low-frequency motions involving conserved hinge and anchor residues in all five channels investigated. The presence of these low-frequency modes in all channel proteins fostered the hypothesis that they reflect a general conformational dynamic in the gating of $\hbox {K}^+$ channel pores.

A critical aspect in coarse-grained modeling is to derive the best possible coarse-grained potential *U* representing a given first principles target potential *V*. To achieve this goal, it is necessary to solve the integral:

1 $$U\left( \mathrm{r}_m\right) = -\frac{1}{\beta } \mathrm {ln} \left[ \int \mathrm {d}\mathrm{r}_t ~ \delta \left( \varvec{\mu }\left( {\bf{r}}_t\right) - {\bf{r}}_m \right) ~ \mathrm {exp} \left( - \beta V({\bf{r}}_t)\right) \right],$$

where $\beta = 1/k_B T$ with Boltzmann factor $k_B$ and temperature *T*. Vectors ${{\bf r}}_t$ and ${{\bf r}}_m$ represent the coordinates of respective entities describing the “particles” of the target and model. $\varvec{\mu }$ corresponds to a mapping $\varvec{\mu }:~\{{{\bf r}}_t\} \rightarrow \{{{\bf r}}_m\}$, defining the coordinates of the coarse-grained model. This serves the purpose of dimensionality reduction, as the high-dimensional vector space of target coordinates is reduced to a lower dimensional space of model coordinates. Typically, Eq. (1) is intractable for realistic system sizes and one has to rely on approximations. While empirically motivated methods like prior chemical knowledge work well for small systems, more sophisticated approaches have to be used for complicated systems.

**Fig. 1:**
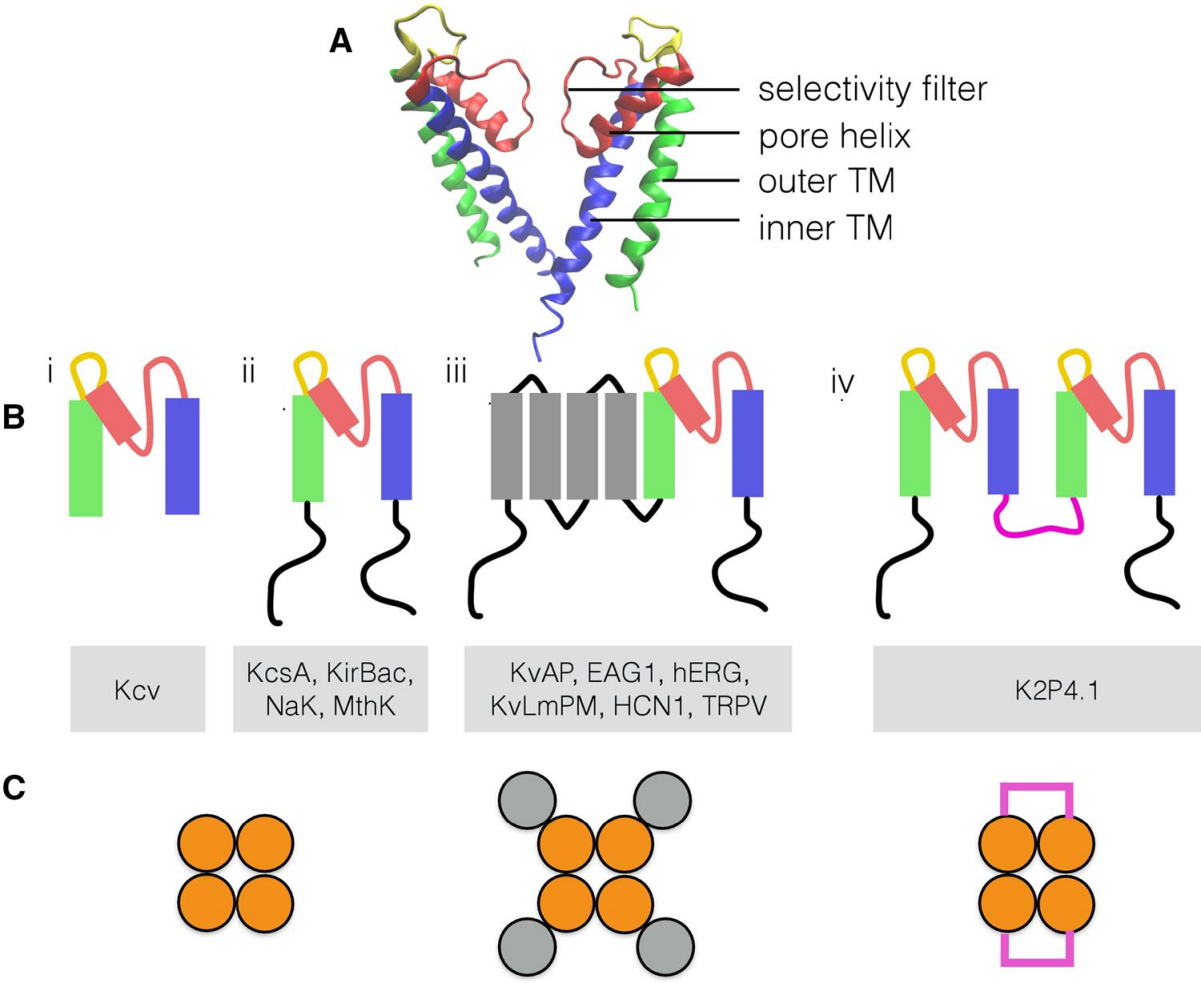
Diversity of $\hbox {K}^+$ channel architectures. (A) The structure of a typical $\hbox {K}^+$ channel pore module is illustrated on the example of two of the four KcsA monomers. The relevant functional domains are color coded with the inner (blue) and outer (green) transmembrane domain, the pore helix plus selectivity filter (red), and the turret (yellow). (B) In functional channels, the pore module can function alone (i), or contain long cytosolic domains (ii). The pore module can also be joined to the voltage sensor domain with four additional transmembrane domains (gray) (iii). Furthermore, two pore modules can be joined by a linker (purple) in a monomer (iv). Channel proteins, which represent the respective architectures and considered in this study, are listed below as respective cartoons. The color code in B, C is the same as in A. (C) In functional channels, the protein structures from B associate in tetramers in which the pore modules i–iii form the central unit; the voltage sensor domain (gray) is peripheral to the central pore. The monomers in which two pore modules are linked (purple) (iv) associate as dimers

Here, we elaborate a protocol for minimizing the relative entropy (Shell 2008) in a protein structure beyond parameter fitting by a second optimization level, which identifies the optimal mapping to a topology with reduced dimension. For this purpose, we consider anisotropic network models of a diverse set of $\hbox {K}^+$ channels and assess our findings by comparison to experimental results. These proteins are, for several reasons, most suited for the present attempt of understanding structure/function correlates by a minimization of the relative entropy. First, the literature provides an increasing number of high-resolution structures of $\hbox {K}^+$ channels and many detailed functional studies, which allow profound analysis of structure and function correlates in channel proteins. Second, $\hbox {K}^+$ channels are modular proteins, which all contain a common pore forming module (Fig. 1A, B). The latter is the functional core unit, which conducts the ions in a channel. Third, functional $\hbox {K}^+$ channels cover a large range of complexity ranging from the most simple isolated pore modules (Fig. 1Bi) to complex channels in which regulatory domains such as the voltage sensor domain or large cytosolic domains are attached to the pore module (Fig. 1Biii,iv).

The structures of the common pore module in each monomer can be dissected into distinct functional domains, which are illustrated in Fig. 1A. The outer transmembrane domain anchors the proteins in the membrane (Kir-type channels, Fig. 1Bi, ii) or interacts with the surrounding transmembrane domains (all other channels, Fig. 1Biii,iv). The inner helix forms a water-filled cavity, which is part of the ionic pathway. The two TMDs are connected via the pore helix and a highly conserved selectivity filter. The latter provides a polypeptide backbone in which the carbonyl oxygen atoms are aligned in such a manner that they substitute the oxygen in the water molecules of the hydration shell of the $\hbox {K}^+$ ion. The positioning of the delicate filter domain is achieved by the pore helix, which forms a hydrogen-bond network with the filter forming amino acids and connects to the outer transmembrane domain via the so-called turret. Interesting for the present analysis is that all channels exhibit the same overall core architecture, while each of them still shows some distinctly different functional features.

The NaK channel (Fig. 1Bii) for example differs in the selectivity filter from the other channels in one amino acid with the result that it conducts not only $\hbox {K}^+$ but also Na$^+$ (Alam and Jiang 2009b). The most primitive among the remaining $\hbox {K}^+$-selective channels is the viral Kcv channel, a channel with no specific regulation of channel gating (Fig. 1bi, (Thiel et al. 2011). The remaining channels (Fig. 1B) in contrast exhibit distinct gating properties. The KirBac3.1 channel functions as an inward rectifier (Cheng et al. 2009), while KscA is activated by acidic pH (Thompson et al. 2008) and MthK by Ca$^{2+}$ (Jiang et al. 2002a). The Kv-type channels (KvAP, HCN1, Eag1, and hERG) are gated by voltage via a distinct voltage-sensing domain (Fig. 1biii) and by different ligands (Gonzalez et al. 2012). The dimeric K2P4.1 channel is selective for $\hbox {K}^+$ despite its different assembly (Fig. 1biv, (Lolicato et al. 2014)), while TRPV1 and TRPV2 (Fig. 1biii) are non-selective and voltage-insensitive cation channels in spite of the fact that their global architecture resembles that of Kv channels (Cao et al. 2013; Huynh et al. 2016).

The present study is motivated by the idea that the similar architecture of the pore domains of these proteins and their common function as ion channel bears information on the most fundamental structure/function correlates in ion channels, which can be extracted from the Kullback–Leibler divergence. This information could deepen our understanding on how $\hbox {K}^+$ channels function and inspire the design of synthetic channel pores. To tackle this problem, we apply our protocol of structural minimization to channel domains with increasing complexity. All considered channels and their respective protein data bank structures are shown in Table 1. We first analyze the structural features, which are common to the pore module (Fig. 1bi,ii). In the next step, we address the question whether these functional features are maintained in more complex channels in which the pore module is connected to other protein domains (Fig. 1biii,iv). With this analysis, we find a set of four critical amino acids in the pore module, which seem to be most important for the function in most K^+^ channels with the same architecture.

**Table 1:** Ion channels used in our study and the respective references. The right column shows the highest scoring residues within the AIC minimization

Channel	pdb code	Total number res. (channel monomer)	Res. considered in analysis (channel monomer)	Highest scoring res.
KcsA (Doyle et al. 1998)	1BL8	173	23-119	71, 76, 105, 110
KcsA (Uysal et al. 2009)	3EFF	173	22-160	71, 76, 96, 101
KcsA (Uysal et al. 2011)	3PJS	173	22-160	71, 76, 95, 100
KcsA (Morais-Cabral et al. 2001)	1JVM	173	24-120	71, 76, 105, 110
KcsA	3F5W	173	30-117	71, 76, 95, 100
KcsA (Lenaeus et al. 2014)	2W0F	173	23-124	76, 105, 110, 115
KcsA (Lockless et al. 2007)	2ITD	173	22-124	100, 105, 110, 115
KcsA (Lockless et al. 2007)	2NLJ	173	22-124	71, 76, 96, 105
KvAP (Lee et al. 2005)	2A0L	295	142-237	194, 217, 222, 228
Eag1 (Whicher and MacKinnon 2016)	5K7L	962	346-479	432, 437, 459, 464
hERG (Wang and MacKinnon 2017)^a^	5VA2	1159	335-457	410, 415, 444, 449
KvLm-PM (Santos et al. 2012)	4H33	139	12-102	53, 58, 78, 83
TRPV1 (Cao et al. 2013)^a^	3J5Q	838	558-671	617, 645, 651, 656
TRPV2 (Huynh et al. 2016)^a^	5HI9	770	521-652	551, 556, 570, 584
K2P4.1 (Lolicato et al. 2014)^a^	4RUF	393	20-283	125, 130, 154, 161,
				234, 239, 265, 270
HCN1, pore region (Lee and MacKinnon 2017)^a^	5U6O	890	291-411	354, 359, 384, 390
HCN1, full channel (Lee and MacKinnon 2017)^a^	5U6O	890	94-586	354, 359, 384, 390
Kcv (Tayefeh et al. 2009)	-	94	1-94	55, 61, 66, 84
KirBac 3.1 (Clarke et al. 2010)	2WLJ	301	35-137	92, 97, 116, 126
MthK (Posson et al. 2013)	4HYO	106	18-99	55, 60, 80, 87
NaK (Shi et al. 2006)	2AHY	104	1-104	59, 64, 84, 91

The optimization settings were four model residues in every monomer (eight for K2P4.1) and a minimum separation along the backbone of $s=4$

^a^Incomplete cryo-EM structures. Missing residues were added as described in Materials and methods

## Materials and methods

We base our study on the relative entropy, also known as Kullback–Leibler divergence $D_{KL}$ in information theory (Kullback and Leibler 1951), as a crucial parameter to derive coarse-grained models. For a target equilibrium probability distribution $p({{\bf r}}_t;\kappa ^{t})$ and model distribution $q(\varvec{\mu }({{\bf r}}_t);\kappa ^{m})$ defined on the same continuous set $\{{{\bf r}}_t\}$:

2 $$\begin{aligned} D_{KL}(p||q)=\int d{{\bf r}}_t~ p\left( {{\bf r}}_t;\kappa ^{t}\right) ~ \mathrm {ln} \left( \frac{p\left( {{\bf r}}_t;\kappa ^{t}\right) }{q\left( \varvec{\mu }\left( {{\bf r}}_t\right) ;\kappa ^{m}\right) } \right) \end{aligned}$$

measures the information-theoretic distance between *p* and *q*. Here, we denote by $\kappa ^{t}/\kappa ^{m}$ the parameters on which the respective target/model distribution depends. The $D_{KL}$ can be used as a loss function to “learn” the model *q* from the known or otherwise accessible “true” model *p*.

$D_{KL}(p||q) = 0$ if and only if $p \equiv q$ for all coordinates but on a vanishing Lebesgue measure. The minimization of Eq. (2) with respect to model parameters $\kappa ^{m}$ leads to a model distribution *q* which best represents the target distribution *p*, i.e., a model that best represents the dynamical and structural properties of the target. This procedure was first applied in (Ming and Wall 2005b) and (Ming and Wall 2005a) for ANMs and in (Chennubhotla and Bahar 2007) for Gaussian network models (GNM). In the context of general coarse-graining, it was made popular by (Shell 2008) and then used extensively from thereon (Saunders and Voth 2013; Noid 2013; Chaimovich and Shell 2010, 2011; Shell 2016). For Boltzmann distributions *p* and *q*, the minimization of Eq. (2) w.r.t. model potential *U* is consistent with the integral in Eq. (1) (see Appendix  5.1 for details).

Minimizing the Kullback–Leibler divergence w.r.t. model parameters $\kappa ^{m}$ corresponds to maximizing the likelihood of reproducing target observations by the model, as:

3 $$\begin{aligned}&argmin_{\kappa ^{m}} D_{KL}(p||q)\nonumber \\&\quad = argmax_{\kappa ^{m}} \int d{{\bf r}}_t~ p({{\bf r}}_t;\kappa ^{t}) ~ \mathrm {ln} \left( q\left( \varvec{\mu }\left( {{\bf r}}_t\right) ;\kappa ^{m}\right) \right) \end{aligned}$$

represents a logarithmic likelihood function.

In combination with model selection procedures like Akaike’s information criterion (AIC) (Akaike 1998), this can be used as a powerful tool for comparison and selection of model parameters and mappings. In particular, maximizing Eq. (3) and using AIC as an evaluation criterion make it possible to compare the ability of different mappings to reproduce the target distribution^1^.

Additionally, if the operator $\varvec{\mu }$ is chosen, such that:

4 $$\varvec{\mu }\left( {{\bf r}}_t\right) = {\bf{s}}_t,$$

where $\mathrm{s}_t$ represents a subset of coordinates $\mathrm{r}_t$ of supposedly important residues, a direct identification of important target residues is possible through the model selection procedure. We give a detailed description of our selection procedure in the following text.

### Optimization scheme

For each considered channel, we start with a $C_\alpha$-based ANM and choose our mapping according to Eq. (4), such that the model residues are a subset of all target $C_\alpha$. We then minimize Eq. (3) w.r.t. the coupling parameters $\kappa ^m$ of a reduced ANM. By a second-level optimization of the concrete mapping $\varvec{\mu }$, we find the mapping with minimum AIC and identify important residues of the target. Furthermore, we are able to find (de)coupled regions in the target ANM by changing the score function of our second optimizer. In the following, we describe the detailed procedure of our approach.

### Anisotropic network model

Elastic network models like GNMs (Tirion 1996), and especially their extension to ANMs (Atilgan et al. 2001), have become an important method for dynamical analyses of proteins (Ikeguchi et al. 2005; Eom et al. 2007; Bahar et al. 2010). Starting with a protein data bank (pdb) structure, the interaction between every residue pair *ij* is described by a harmonic potential with coupling constant $\kappa _{ij}$. The couplings represent our target ($\kappa _{ij}^t$) or model ($\kappa _{ij}^m$) parameters, where we minimize Eq. (2) in the latter. Performing a Taylor expansion of the first-principle potential up to second order around the equilibrium configuration (pdb structure) ${{\bf r}}^0$ yields the potential:

5 $$\begin{aligned} V_\mathrm {ANM}({{\bf r}})=\frac{1}{2} \mathrm{\bf r}^T \cdot H \big \vert _{{{\bf r}}^\mathrm {0}} \cdot \mathrm{\bf r}, \end{aligned}$$

where $\mathrm{r}$ denotes the displacement from the equilibrium configuration. The Hessian $H := H\big \vert _{{{\bf r}}^\mathrm {0}}$ is composed of (3 × 3) submatrices $h_{ij}$, which for $i \ne j$ reads:

6 $$\begin{aligned} h_{ij} = \frac{\kappa _{ij}}{(s_{ij}^\mathrm {0})^2} \begin{bmatrix} x_{ij}^0 x_{ij}^0 &{} x_{ij}^0 y_{ij}^0 &{} x_{ij}^0 z_{ij}^0\\ y_{ij}^0 x_{ij}^0 &{} y_{ij}^0 y_{ij}^0 &{} y_{ij}^0 z_{ij}^0\\ z_{ij}^0 x_{ij}^0 &{} z_{ij}^0 y_{ij}^0 &{} z_{ij}^0 z_{ij}^0 \end{bmatrix} \end{aligned}$$

with $s_{ij}^\mathrm {0}$ being the equilibrium distance between *i* and *j* and $x_{ij}^0$, $y_{ij}^0$ and $z_{ij}^0$ being the differences in the corresponding directions. The diagonal elements are given by $h_{ii}^{(a,b)} = -\sum _{j,j\ne i} h_{ij}^{(a,b)}$, where *a* and *b* denote the scalar entry.

In the following, we consider a target ANM with $H_t$ and a model ANM with $H_m$, where both matrices have equal dimensions (3*n* × 3*n*). For the construction of $H_t$, we take the coordinates ${{\bf r}}^0_t$ of all *n* $C_\alpha$ atoms in the pdb and use the bio3d package (Bj et al. 2006) in R (R Development Core Team 2008) with residue and distance specific couplings $\kappa _{ij}^t$ from (Dehouck and Mikhailov 2013). For the construction of $H_m$, we take a subset $\mathcal {M} \subset \{1,...,n\}$ of the $C_\alpha$ atoms and initialize all model couplings with $\kappa _{ij}^m =1$. All submatrices $h_{ij}$ of $H_m$ with $i,j \in \{1,...,n\}~\wedge ~\left( i \notin \mathcal {M}~\vee ~ j \notin \mathcal {M}\right)$ are filled with zeros and the corresponding couplings are not considered in the following optimization. The Hessians $H_t$ and $H_m$ are arranged in such a way that the residues at the *ij*th position correspond to each other, i.e., every *ij* pair denotes the same residue combination in both Hessians.

Both Hessians have at least six zero eigenvalues due to rotational and translational symmetries ($H_m$ much more, depending on the size of $\mathcal {M}$). Our further calculations require the invertibility of $H_{t}$ and $H_{m}$ (see, e.g., Eq. 10), and thus, we slightly perturb them by $H_{t/m} \rightarrow H_{t/m} + \epsilon \mathbb {1}$ on the diagonal via the identity matrix $\mathbb {1}$. Typically, $\epsilon \le 0.001$, and therefore, the reduced model depends (almost) only on those residues *i* in ${{\bf r}}_t$, for which $i \in \mathcal {M}$, fulfilling Eq. (4).

Some of our considered ion channels had incomplete pdb structures. For the construction of the ANM, the missing residues were added using the autoloop function of Modeller 9.19 (Fiser et al. 2000). Depending on the channel architecture, 2- or 4-fold symmetry of the modeled regions was enforced on the backbone atoms.

### Minimization of Kullback–Leibler divergence

Given the above Hessians, we can define the corresponding target and model Boltzmann distributions:

7 $$\begin{aligned} p\left( {{\bf r}}_t\right) :=&\frac{1}{Z_p} \mathrm {exp} \left[ -\beta V({{\bf r}}_t) \right] = \frac{1}{Z_p} \mathrm {exp} \left[ -\beta \frac{1}{2} {{\bf r}}^T_t \cdot H_t \cdot {{\bf r}}_t \right] , \end{aligned}$$

8 $$\begin{aligned} q({{\bf r}}_t) :=&\frac{1}{Z_q} \mathrm {exp} \left[ -\beta U({{\bf r}}_t) \right] = \frac{1}{Z_q} \mathrm {exp} \left[ -\beta \frac{1}{2} {{\bf r}}^T_t \cdot H_m \cdot {{\bf r}}_t \right] , \end{aligned}$$

with $Z_p$ and $Z_q$ denoting the partition functions. Putting above distributions into Eq. (2) yields:

9 $$\begin{aligned} D_{KL}\left( p || q\right) = \frac{1}{2} \mathrm {tr}\left[ H_m (H_t)^{-1} \right] - \frac{1}{2} \sum _{i=1}^{3n} \mathrm {ln}\left( \lambda _i^m \right) + \frac{3n}{2} \mathrm {ln}\left( \frac{2 \pi }{\beta } \right) - S_t, \end{aligned}$$

where $\lambda _i^m$ denotes the eigenvalues of $H_m$ and $S_t$ the entropy of the target distribution. The first three terms correspond to the negative logarithmic likelihood for reproducing target observations by the model, while only the first two terms depend on model parameters $\kappa _{ij}^m$. The derivatives of the first two terms are given by:

10 $$\begin{aligned} \frac{\partial D_{KL}\left( p || q\right) }{\partial \kappa _{ij}^m}= \frac{1}{2} \mathrm {tr}\left\{ \left[ (H_t)^{-1} - (H_m)^{-1} \right] \frac{\partial H_m}{\partial \kappa _{ij}^m} \right\} . \end{aligned}$$

Furthermore, $D_{KL}(p || q)$ is convex in $\kappa _{ij}^m$ (Bilionis and Zabaras 2013), as the Hessian:

11 $$\begin{aligned} \frac{\partial ^2 D_{KL}(p || q)}{\partial \kappa _{ij}^m \partial \kappa _{kl}^m}= & {} \frac{\beta ^2}{4} \cdot \left\{ \left\langle {{\bf r}}_t^T \frac{\partial H_m}{\partial \kappa _{ij}^m}{{\bf r}}_t \cdot {{\bf r}}_t^T \frac{\partial H_m}{\partial \kappa _{kl}^m}{{\bf r}}_t \right\rangle _{q\left( {{\bf r}}_t\right) }\right. \nonumber \\&\left. - \left\langle {{\bf r}}_t^T \frac{\partial H_m}{\partial \kappa _{ij}^m}{{\bf r}}_t\right\rangle _{q({{\bf r}}_t)} ~ \left\langle {{\bf r}}_t^T \frac{\partial H_m}{\partial \kappa _{kl}^m}{{\bf r}}_t \right\rangle _{q\left( {{\bf r}}_t\right) } \right\} \end{aligned}$$

has the same structure as a covariance matrix, which is always positive semidefinite.

We implemented the above optimization in the julia language (Bezanson et al. 2017) using the NLOPT library (Johnson 2007-2020) for gradient-based optimization. The average runtime for $|\mathcal {M}| = 16$ and a protein length of 300 residues is less than one second on a single CPU-core (Intel(R) Core(TM) i7-6700 CPU @ 3.40GHz). To restrict ourselves to physically relevant solutions, we constrained $\forall i,j:~\kappa _{ij}^m > 0.1$. As $\kappa _{ij}^m > 0.1$ is enforced, the chosen epsilon of $\epsilon \le 0.001$ will not influence the overall results substantially.

### Secondary optimization

We perform a second optimization in the mapping $\varvec{\mu }$ by simulated annealing (Kirkpatrick et al. 1983). Here, we take into account both the number of model residues $|\mathcal {M}|$ as well as their corresponding positions $i \in \{1,...,n\}$. We consider three different scoring functions $f^\mathrm {sc}$:

#### Finding the best representative model

To find the best representative model, we use:

12 $$\begin{aligned} f^\mathrm {sc} = -\mathrm {AIC}=2\mathrm {ln}(\hat{L})-2k \end{aligned}$$

as an evaluation criterion (Akaike 1998). $f^\mathrm {sc}$ is the negative of the Akaike criterion (to always be faced with a *maximization* problem, see below)^2^. Here, *k* denotes the number of model parameters (i.e., the number of distinct $\kappa _{ij}^m$) and $\hat{L}$ the value of the optimized likelihood function, in this case calculated by Eq. (3). Without changing the result of the minimization process, we use the first two terms of (minimized) Eq. (9) for $\hat{L}$, neglecting the constant third and fourth terms that do not depend on the minimization variables $\kappa _{ij}^m$. The AIC implies a trade-off between the quality of fit and the number of model parameters.

#### Finding strongly coupled residues

To find strongly coupled residues, we use the $p=\frac{1}{2}$ norm:

13 $$\begin{aligned} f^\mathrm {sc}_{+} = ||\kappa ^m||_{\frac{1}{2}}= \left( \sum _{i<j} \sqrt{\kappa _{ij}^m} \right) ^{2} \end{aligned}$$

as a scoring function. The square root in the sum ensures that every coupling has a high value (in contrast to, e.g., a square which can favor low couplings as long as there is a small set of high couplings).

#### Finding decoupled residues

To find decoupled residues, we use the negated $p=2$ norm:

14 $$\begin{aligned} f^\mathrm {sc}_{-} = - ||\kappa ^m||_2 = - \sqrt{\sum _{i<j} (\kappa _{ij}^m)^2} \end{aligned}$$

as the scoring function to be maximized. The square in the sum ensures that every coupling has a low value (as it penalizes even single high values).

**Fig. 2:**
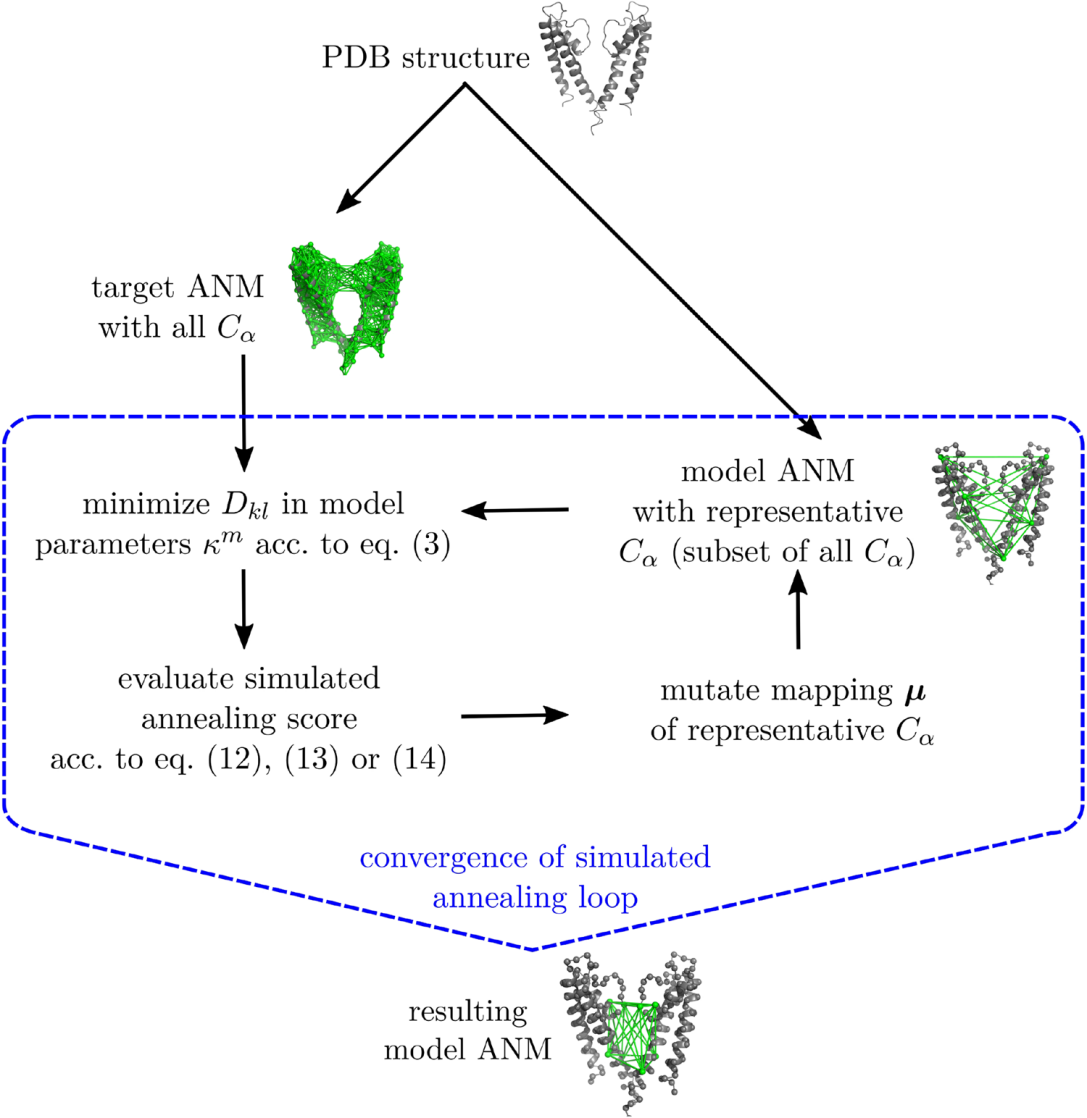
Flowchart of our procedure. Based on a pdb structure, we build a target ANM consisting of all $C_\alpha$ and a model ANM consisting of a (random) $C_\alpha$ subset. In the subsequent simulated annealing loop, we iterate over modifications of the $C_\alpha$ subset. After convergence, we obtain a model ANM that (locally) optimizes our chosen score function

Algorithm 1 shows our complete procedure in pseudocode. For the random selection and modification of representative (model) residues $i,j \in \mathcal {M}$, we choose a minimum separation along the backbone of $|i-j| > 4$. The modification consists of randomly changing a single representative residue *i*. In addition, a flowchart of the above steps^3^ is presented in Fig. 2. 
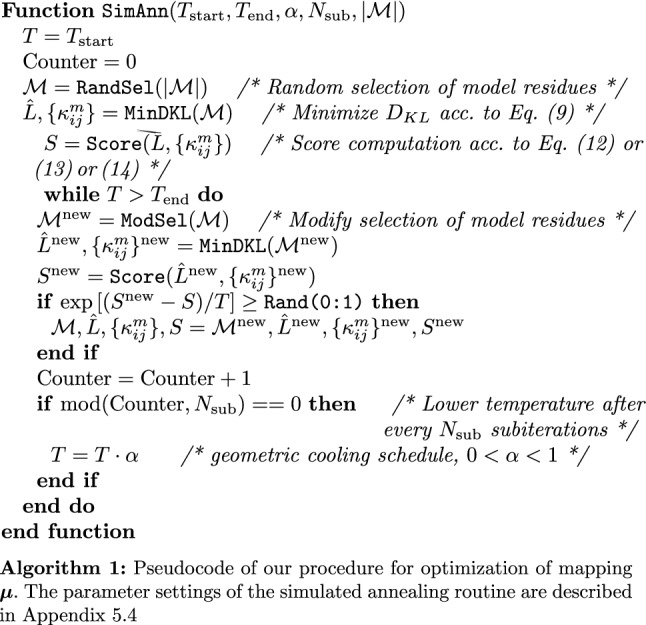

### Symmetry in model residue selection

In our application, all considered channels (except for K2P4.1^4^) are homotetramers with $n=4l$ amino acids (*l* per monomer, see Fig. 1). We accordingly chose our model residues with a fourfold symmetry. This means that if, e.g., a chosen residue *i* is located in the first monomer, we also select the corresponding residue in the other three monomers: $i\in \mathcal {M} \Rightarrow i+l,i+2l,i+3l \in \mathcal {M}$.

The concrete selection and modification of model residues was done by the following random procedure: For the initial selection (RandSel routine in Algorithm 1), we determined each chosen residue *i* by draws from a uniform distribution between 1 and *l*. In case that the desired minimum backbone separation was not satisfied, we repeated this step until convergence. To fulfill our symmetry constraint, we afterwards chose the corresponding residues in the other monomers ($i+l$, $i+2l$, $i+3l$). For the mutation step in the simulated annealing routine (ModSel in Algorithm 1), we randomly selected a model residue *i* and subsequently draw from a uniform distribution (between 1 and *l*) to determine its new position *j*. In case that the desired backbone separation between the new position *j* and any other model residue was not satisfied, this step was repeated until convergence. Afterwards, we again chose the corresponding residues symmetric in the other monomers ($i+l$, $i+2l$, $i+3l$).

## Results

In the following sections, we evaluate first the model parameters and the robustness of the method by comparing several structures of the model channel KcsA. We then perform calculations with the pore modules of a library of structures ranging from simple structures with two transmembrane domains (Fig. 1, bi,ii) to complex potassium channels (Fig. 1, biii,iv) (Table 1) and discuss functional implications of our findings. Finally, we investigate highly mechanically coupled and uncoupled regions in ion channels.

### The anisotropic network of KcsA can be reduced to four amino acid positions

As a test case, we started the analysis with the pore module of KcsA, the best studied $\hbox {K}^+$ channel pore. The mechanical connections in this structure can be represented by the aforementioned ANMs. The edges in such a network represent physical interactions in a protein, which, in turn, are reduced to harmonic interactions between all residues closer than a critical cut-off distance. Previous studies had shown that analyses of these network models are able to generate global maps for the mechanical connections in these proteins (Shen et al. 2002; Shrivastava and Bahar 2006; Hoffgaard et al. 2015). All this should provide information on interactions between functional domains, which are essential for the function of these channel pores.

In the first application, we ask the question whether it is possible to reduce the network models to an even smaller size without compromising their main functional features. For this purpose, we minimize the AIC value (which is equivalent to maximization of $f^\mathrm {sc}$) according to Eq. (12) for models of decreased size. The AIC value quantifies the trade-off between the number of fitting parameters and accuracy of the fitting results. Since KcsA is a homotetramer with 4*l* amino acids (*l* per monomer), we were choosing the model residues with a fourfold symmetry (see Sect. 2.5).

**Fig. 3:**
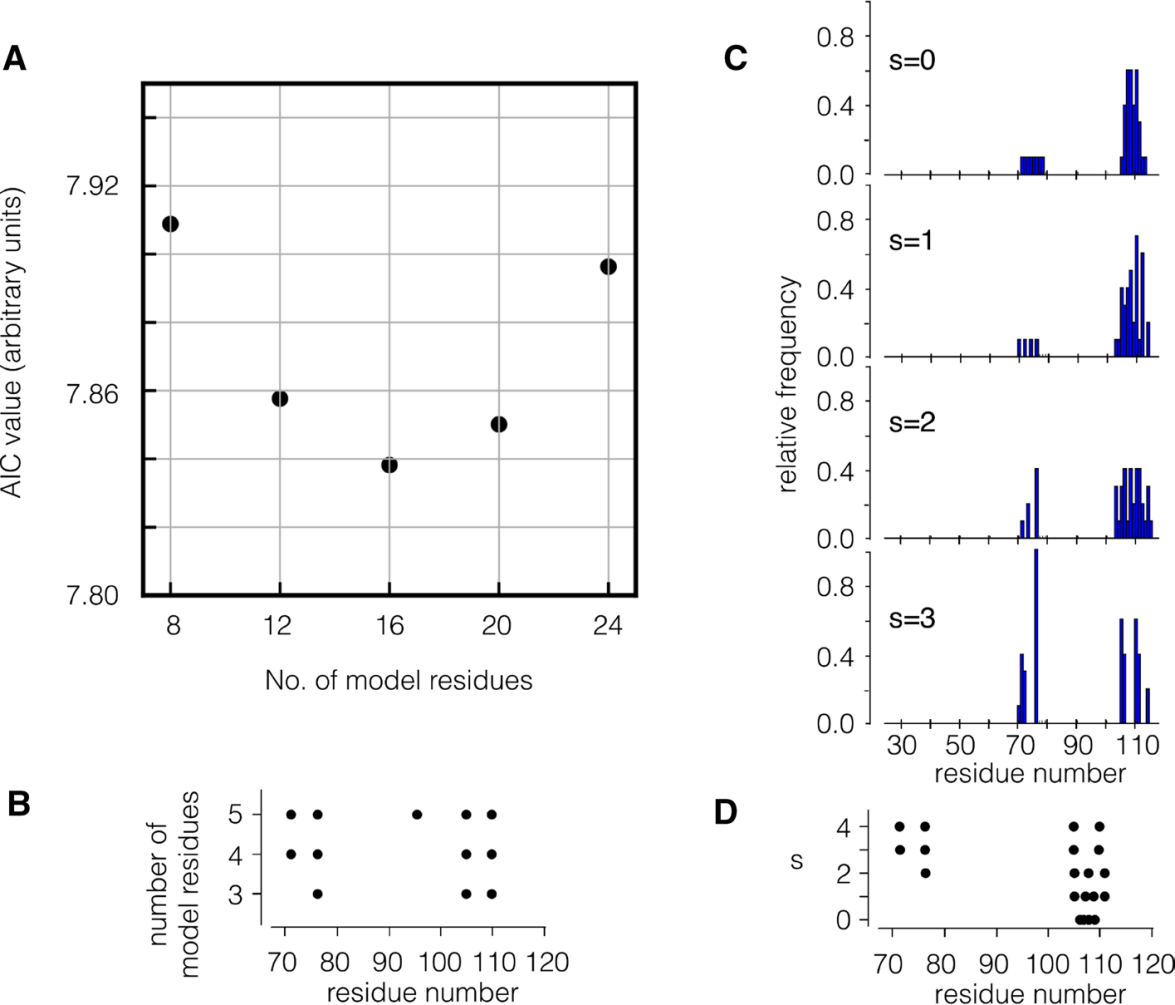
**a** AIC value of KcsA (1BL8) for different numbers of model residues. The AIC exhibits a minimum for 4 model residues in every monomer. **b** Highest scoring residues within AIC minimization for the different numbers of model residues. **c** Identification of critical residues in KcsA structure for 16 model residues under the constraint that individual residues must be separated between $s= 0$ and $s=4$ residues along the backbone. The plots show the relative frequencies of residues with the 10 best AIC values for $s=0$ to $s=3$ for a single monomer. The frequencies were obtained by the construction of a histogram over the identified residues within the 10 highest scores (of all concatenated individual simulated annealing runs, see Appendix 5.4) and a subsequent normalization (division by 10), such that the frequency of every residue lies between 0 and 1. **d** reports top scoring residues as a function of *s*

The analysis shows that the lowest AIC value is obtained with a value of $|{\mathcal{M}}| = 16$, i.e., 4 residues for each monomer. A representative plot which shows a minimum of the AIC value for 16 residues in the KcsA tetramer is shown in Fig. 3a. In Fig. 3b, we exemplify the stability of those “cluster assignments”. For this, we consider AIC minimization with a different number of model residues $|{\mathcal{M}}| \in \{8,12,16,20,24\}$ (which correspond due to the fourfold symmetry to 2, 3, 4, 5, 6 clusters, respectively). Our results show that the identified residues for $|{\mathcal{M}}| = 12$ are a subset of the identified residues for $|{\mathcal{M}}| = 16$, which are again a subset of the identified residues for $|{\mathcal{M}}| = 20$. These findings imply a stability of the AIC minimization for a varying number of model residues. Supplement Table 2 shows the same calculation performed on the other channels with a Kir-type architecture. As for KcsA, a total number of $|{\mathcal{M}}| = 16$ model residues have the lowest AIC value in all channels.

**Fig. 4:**
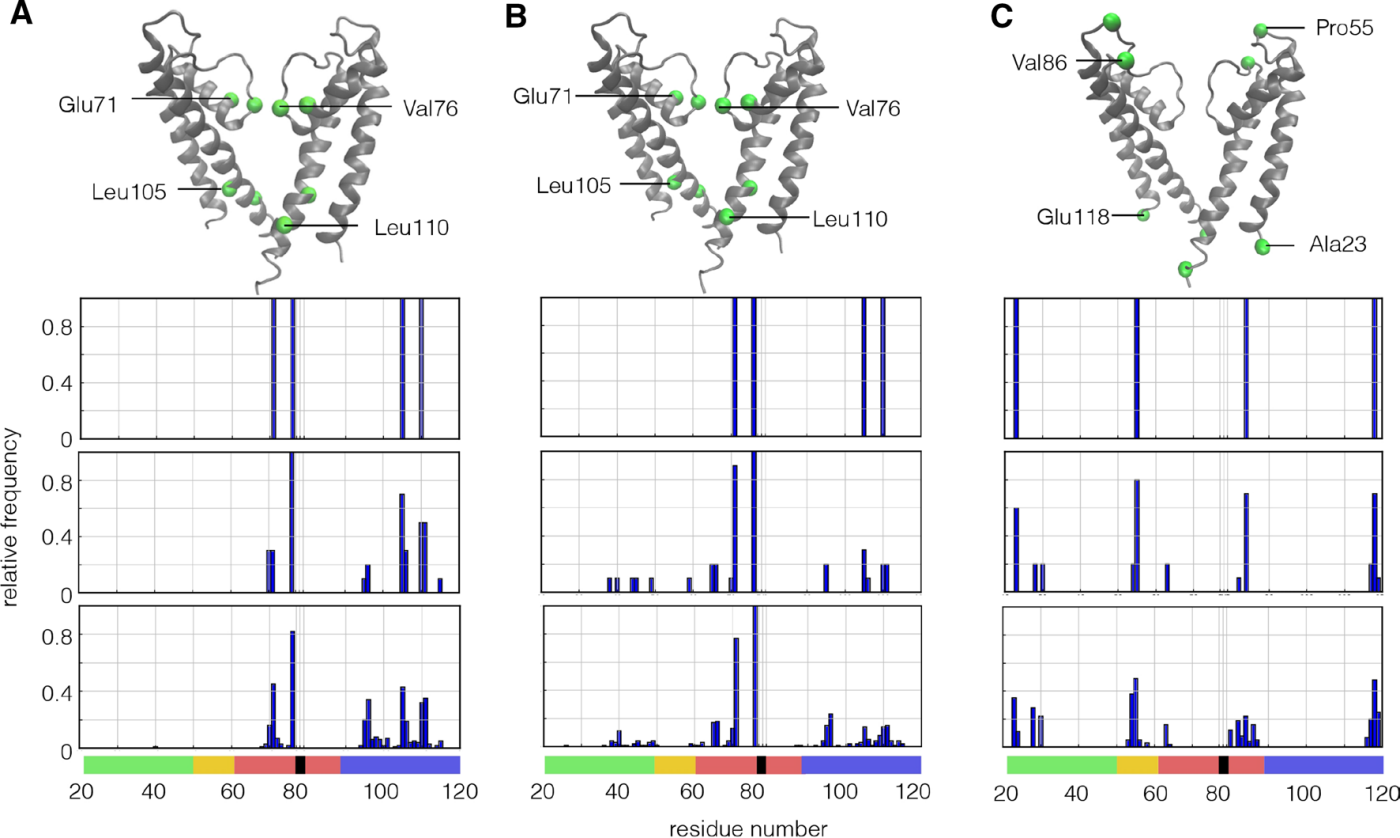
Identification of residues, which are critical in KcsA structure (pdb 1BL8). Two monomers of KcsA channel with localization of 4 critical residues (green spheres) by (**a**) using AIC value (Eq. 12), (**b**) maximal coupling norm (Eq. 13), or (C) minimal coupling norm (Eq.  14) as score value. The residues in A indicate the most important sites for the function of the protein, the residues in **b** indicate the sites with the strongest coupling, and **c** shows the residues, which are most uncoupled. Distributions of the respective residues are shown below the structures with respect to their position in a KcsA monomer. The best scores for each analysis are shown in the first row. The relative frequencies of residues with the 10 and 100 best score values are reported in the second and third row, respectively. The frequencies in every diagram were obtained by the construction of a histogram over the identified residues within the 10 or 100 highest scores and a subsequent normalization. The color bars below the diagrams denote the respective sequence position and are consistent to Fig. 1a. The position of the GYG motive of the filter is indicated by black bars and three vertical lines

The critical residues in the KcsA structure in Fig. 3a,b were calculated with the constraint that they must be separated by more than 4 amino acids. This criterion was introduced to lower the importance of mechanical connections, which are determined by covalent bonds in the protein backbone; the $\alpha$-helices determining the TMDs are characterized by consecutive $i+4$ interactions along the backbone. To test the impact of this constraint on the general results of the calculation, the same procedure was repeated with the KcsA channel in Fig. 3c using a minimum separation along the backbone of $|i-j| > s$ with $s \in \{0, 1, 2 , 3\}$. The results show that a value of $s \le 2$ generates clusters of residues in the inner transmembrane domain, which include the cluster of critical residues discovered with a value of $s=4$ (Fig. 3d). An increase in the s values to 3 lowers the impact of direct amino acid interactions and uncovers essentially the same 4 residues that were identified in the analysis of Fig. 4a. From these control calculations, we conclude that the constraint of $s \ge 4$ guarantees the discovery of critical residues in the entire protein.

### Location of critical amino acids in pore module is insensitive to presence or absence of cytosolic C-terminus

Figure 4a shows the location of the critical four residues in the pore module of the KcsA channel (1BL8). The respective amino acids are 76 in the filter, 71 in the pore helix, and 105 and 110 in the inner transmembrane domain (Table 1). Before evaluating functional implications of the critical residues, we first address the question whether the results are depending on the length of the sequence. Notably, most of the pores, which are evaluated here, are truncated structures from more complex channels (see Table 1).

**Fig. 5:**
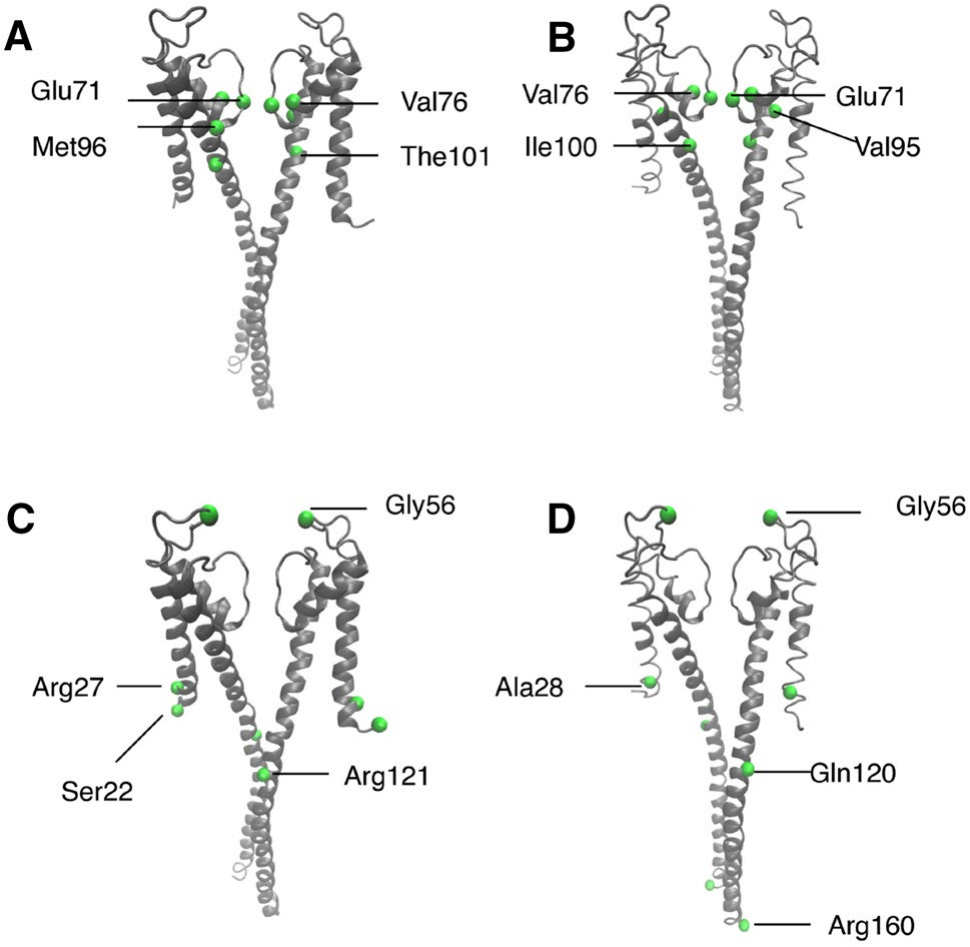
The critical four residues in KcsA structures with extended C-terminus. Residues obtained from minimal AIC values (**a**, **b**) and minimal coupling norm (**c**, **d**) in KcsA structures with extended C-terminus (pdb 3EFF left panel and 3PJS right panel). The residues of interest are depicted in the structures as green spheres

To test the influence of the length of the structure on the critical residues, we performed the same analysis as in Fig. 4 with two KcsA structures, which include the entire C-termini (Fig. 5). This analysis highlights the same residues in the filter domain and similar residues in the inner transmembrane domain as in the reference structure (Fig. 4). The results of this analysis confirm the robustness of the approach. They furthermore suggest that the residues 71 and 76 in the filter domain have a unique importance for function. The residues in the transmembrane domain seem less precisely defined and are presumably more a representation of small critical regions than individual amino acids. The data further imply that the detection of the critical residues is not an artifact of the truncation of the structure.

In the analyses so far, we have only considered four residues with the best score value. The aforementioned data, however, imply that the precise position of the four critical residues can be variable. To judge the significance of these four individual residues, we plotted a normalized histogram of the four residues within the 10 and 100 best AIC score values in Fig. 4a. The data show that the aforementioned four residues occur with a different frequency. While V76 emerges in all cases as a single residue, the other three residues are the peak values of small regional maxima. This indicates that V76 has a unique importance for the function of the KcsA channel. The other residues seem to represent small hot spots, which are important for the function of the KcsA channel. In the pore helix, also the residue V70 seems to be important for function, together with E71. In the inner TM domain, we identify a small cluster of residues around amino acid (AA) M96 and V95 and two clusters around L105 and L110.

### The predictions are robust and most critical residues are discovered in different KcsA Structures

**Fig. 6:**
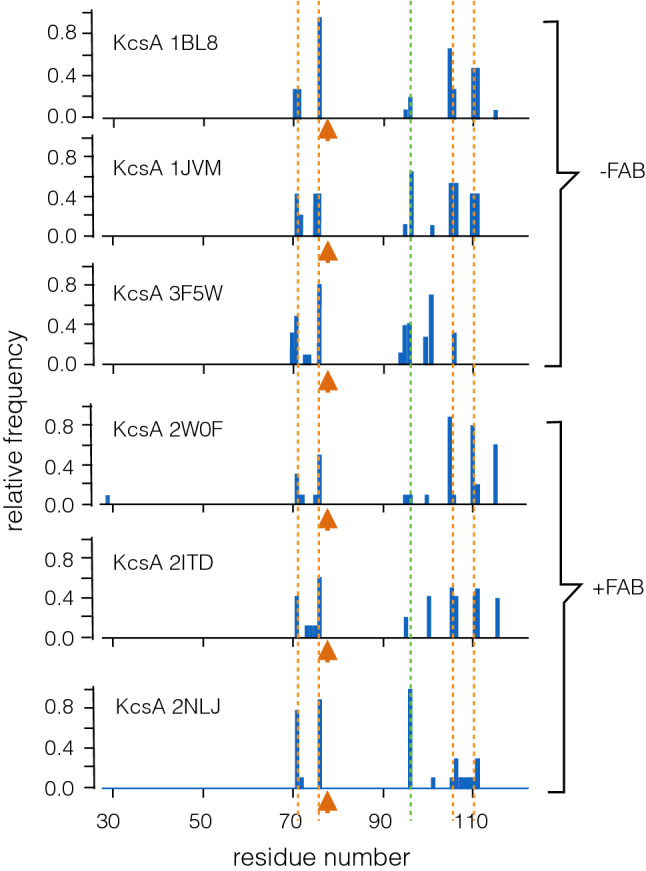
Relative distribution of critical residues in 6 different KcsA structures. Same analysis as in Fig. 4 reports the relative frequencies of residues with the 10 best AIC score values. The plots are aligned to the GYG sequence in the selectivity filter (orange arrow). The dotted lines highlight positions of critical residues, which are observed in all structures. The dotted green line indicates an additional position at residue 96. Different KcsA structures were determined without (1BL8, 1JVM, 3F5W) or with FAB fragments (2W0F, 2ITD, 2NLJ). Other specific features of the different structures are as follows: 1JVM, with tetrabutylammonium; 3F5W, KcsA in an open inactivated state. 2W0F, with tetraoctylammonium; 2ITD with $\hbox {BaCl}_2$; 2NLJ, M96V mutation and with the filter in collapsed configuration

In a further step, we analyzed the robustness of the predictions with different KcsA structures. Calculations as in Fig. 4a were repeated with structures obtained under different experimental conditions, e.g., in the absence and presence of a blocker or from KcsA mutants (Table  1). Another difference was that the proteins were crystallized either without or with a antigen-binding fragment (FAB) (Fig. 6). A comparison of the results should imply whether a monoclonal FAB fragment, which supports crystallization by mechanically stabilizing the protein, has any influence on the mechanical connectivity in the channel. The results in Fig. 6/Table 1 show that the critical residues in the filter, which were identified in Fig. 4a, are discovered with high propensity in all KcsA structures. The results of this analysis underscore that these residues seem to be most critical for the functional mechanics of the channel. This appears to be independent of a block of the pore for example by Ba$^{2+}$ (pdb 2ITD) or quaternary ammonium compounds (pdb 1JVM, 2W0F). Also the presence or absence of the FAB fragments has no appreciable impact on the mechanical connectivity of the KcsA pore.

Scrutiny of the remaining residues reveals that they are all located, independent from the crystallization conditions, in the inner transmembrane domain. Their precise localization is more variable than those in the filter domain. This result can already be anticipated from the data in Fig. 4a, which suggests the importance of small clusters of residues rather than of individual amino acids. Independent on whether the structures were obtained with or without FAB fragments, the analysis highlights the crucial importance of residues 105 or 110 and their direct vicinity. The two exceptions are found in the pdbs 3F5W and 2NLJ. In the former structure, the critical residues in the transmembrane domain occur further upstream. In the latter structure, the residues around 105 and 110 are found with a lower propensity, while the maximum is shifted to residue M96, a residue, which shows up with a lower frequency in all other KcsA structures. This finding is very interesting, because the channel structure of pdb 2NLJ was obtained from a KcsA mutant in which the critical M96 was mutated into an V (Lockless et al. 2007). In this channel, the filter remains in a collapsed configuration even in high $\hbox {K}^+$ concentrations. It is tempting to speculate that our analysis is picking up this change in the mechanics of the KcsA channel, which is imposed by a mutation of this critical residue.

### The same critical amino acid positions, which are identified in KcsA, are also relevant in other $\hbox {K}^+$ channel pores

**Fig. 7:**
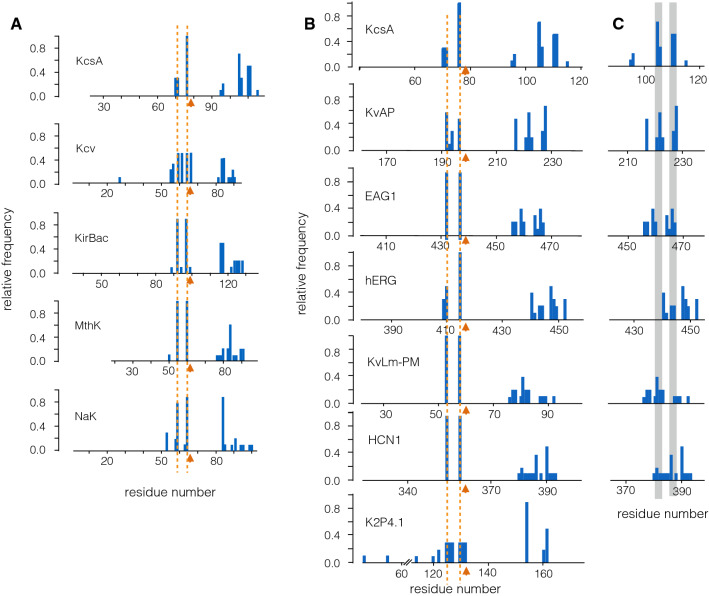
Relative distribution of critical residues in pores of a variety of channel structures. Same analysis as in Fig. 4 for four channel proteins with a Kir-type channel architecture (**a**; Kcv, KirBac, MthK, and NaK) and for 5 pores from complex Kv-type channels (KvATP, Eag1, HCN1, hERG, KvLm-PM), and one K2P channel (K2P4.1) (**b**). The plot shows the relative frequencies of residues with the 10 best AIC. The data from KcsA (pdb 1BL8) are shown in top row for reference. The plots are aligned to the GXG sequence in the selectivity filter (orange arrow). The dotted lines highlight positions of critical residues, which are observed in all structures. Due to consistency reasons, only the first four (out of eight) critical residues are plotted for K2P4.1. (**c**) Plot with critical residues in inner transmembrane domain (=S6 in Kv channels) from B were aligned to start of respective $\alpha$-helix (residue 86 in KcsA, 207 in KVAP, 451 in EAG, 430 in hERG, 73 in Kvlm, and 370 in HCN1). The dominant clusters in KcsA are marked by gray bars

In a next step, we performed the same analysis to a set of other channel pore modules (from KirBac3.1, NaK, MthK, Kcv) which all have the same architecture as KcsA (Fig. 1b, c). An interesting test case is Kcv, because this miniature channel comprises not more than the pore module of all other $\hbox {K}^+$ channels. Even though there is no experimental structure available for this channel, we still considered it for the analysis, because homology models exhibit spontaneous ion translocation in MD simulations (Tayefeh et al. 2009; Andersson et al. 2018). The remaining pores are from Kir-type channels, which have in addition more or less long cytosolic domains (Fig. 1). When we used the same analysis, which was performed on KcsA, on these channels, the obtained 10 highest scores show very similar results (Fig. 7; left panel).

With the exception of the Kcv channel, we found in all cases a singular peak for the residue, which is equivalent to V76 in KcsA in all other channels. Also the equivalent of E71 in KcsA was detected in all other channels with a high frequency. The results of this analysis underscore the unique importance of a residue in the selectivity filter of all channel pores, which is equivalent to the V76 position in KcsA. Also important are residues in the pore helix, which are equivalent to V70 and E71 in KcsA. The situation is more variable in the inner TM domain. All channels contain in this domain clusters with important residues. However, they appear with different frequencies and at different positions relative to the selectivity filter. For example, while the KirBac channel has a small cluster of residues with a low frequency approx. 15 amino acids (AA) downstream of the GYG motive, the critical AA in KcsA are ca. 30 AA away of this domain.

The results so far underpin that the pore modules of $\hbox {K}^+$ channels, which arrange as tetramer with 2 transmembrane domains, have a common functional architecture with four critical amino acid positions, two in the selectivity filter and two at the end of the inner transmembrane domain. The position of these critical residues is not identical in different channels but in the same region of the channel. The channels, which were used for this analysis, exhibit different degrees of selectivity for $\hbox {K}^+$ over Na$^+$ (e.g., KcsA versus NaK) and are gated by different mechanisms (e.g., KcsA versus MthK). The finding that they all share the same critical residues suggests that these positions in the protein are responsible for general functional features but not for selectivity or gating.

### The critical amino acid positions are relevant for function

A scrutiny of the rich literature on structure/function correlates in the KcsA channel confirms the importance for the four crucial amino acids, which emerged from our analysis. All four amino acids turned out in a number of experimental and computational studies as key amino acids for function. The E71 residue in the pore helix has been described as a crucial part of a complex hydrogen-bond network between the pore helix and the selectivity filter. It involves the side chains of E71, D80, and W67, a water molecule, and the backbone atoms of G77 and Y78. Mutations of residues in this network affect the central functions of the channel, namely ion selectivity (Cheng et al. 2011) and gating (Cordero-Morales et al. 2011). Additionally, also the tetramer stability is affected (Choi and Heginbotham 2004). A well-studied mutation in this context is E71A, which suppresses an inactivation process similar to C-type inactivation in KcsA.

The amino acid Val76 in the selectivity filter is mentioned in a number of papers as an important amino acid for proper folding of the KcsA channel (Splitt et al. 2000; Raja and Vales 2009). But also channel function is sensitive to changes in this position. A mutant of KcsA, which imitates the filter sequence of Kir channels (V76I), shows a lower unitary conductance and reduced open probability (Raja and Vales 2009). This stresses an impact of this site on both key functions of channels, namely, conductance and gating. Mechanistic insight for the function of V76 in the KcsA channel comes from the analysis of NMR spectra of the full-length KcsA channel in the activated and inactivated state (Imai et al. 2010). From these data, it occurs as if V76 undergoes a large chemical shift when the channel switches between the resting state, the activation state, and the inactivation state. This has been interpreted as evidence for the importance of this amino acid in filter gating. It has been speculated that this gating is due to a formation of hydrogen bonds between V76 and water and a consequent removal of $\hbox {K}^+$ from the filter (Imai et al. 2010).

The amino acid L110 and its immediate neighbors in the inner TMD of KcsA have been identified in computational (Shen et al. 2002; Shrivastava and Bahar 2006) and experimental studies (Liu et al. 2001; Sompornpisut et al. 2001) as a crucial amino acid in channel function. It is part of a pivot region for inter-subunit interactions formed by a stretch of AAs from Thr107 to Leu110. In this domain, in particular, L110 makes in the truncated KcsA structure an inter-unit contact between the inner TMDs in KcsA (Minor et al. 1999). Computational data including normal-mode analysis suggest that this region is undergoing a low-energy deformation in the context of the operation of the inner gate. This gating motion is discussed in the context with a kink that occurs at Thr 107 in KcsA and which is conserved in KirBac as Gly134 (Shrivastava and Bahar 2006).

Also, the AA L105 is mentioned in the context of KcsA function albeit less frequently than the three other AA. One piece of evidence for its importance in KcsA function is derived from the aforementioned NMR analysis, which reports the chemical shift between the resting state, and the active and inactive states of this channel (Imai et al. 2010). In the NMR spectra, it occurs that the chemical shift of V76 with its impact on filter gating is correlated with that of L105 and V95. Interesting to note is that the plots in Figs. 4a and 7a show an additional peak at residues around V95 (see also Supplement Fig. 9), suggesting that this region is part of a functional hot spot for KcsA gating (Shen et al. 2002; Imai et al. 2010). This may suggest a concerted action of residues in the inner TM and the selectivity filter in channel gating. Other evidence for a role of residue L105 and its vicinity in KcsA function comes from a genetic selection of KcsA mutants with a gain of function. All these mutations were clustering in the helix bundle crossing of the inner TMD including mutations in L105 (Paynter et al. 2008). The functional impact of this amino acid may once again depend on the fact that the inner TMD is involved in subunit–subunit interactions in which Ala29 on the first outer TMD is interacting with L105 and V106 in the inner TMD (Williamson et al. 2002).

A comparison of the critical residues between the different channels (Fig. 7, Supplement Fig. 9) shows that they are, with the exception of the Val in the selectivity filter, not formed by conserved amino acids. This suggests that the proteins have retained throughout evolution a common architecture rather than individual amino acids. A combination of the present results and experimental data from the respective channel pores advocates two architectural principles, which characterize these proteins. One structural feature seems to be a stabilization of the filter domain by an interaction of the pore helix with the selectivity filter. Experimental evidence for these interactions is available for all the channels (Miloshevsky et al. 2008; Shen and Guo 2009; Rauh et al. 2018). The second conserved building principle seems to be a flexibility of the lower part of the inner transmembrane domain. The critical cluster(s) of residues in this domain in all channels (apart from Kcv) are close to a Gly residue (Fig. 7, Supplement Fig. 9). This AA and its neighboring residues have been identified in many channels as a key hinge for promoting flexibility in the lower part of this domain (Jiang et al. 2002b; Rosenhouse-Dantsker and Logothetis 2006; Grottesi et al. 2005; Alam and Jiang 2009a). Interesting to note is that the same functional principle is maintained in Kcv. Only in this channel, the transition from a rigid upper part to a flexible lower part of the transmembrane domains is achieved by a $\pi$-stacking between a His in the inner and a partner amino acid in the outer transmembrane domain (Gebhardt et al. 2011). The cluster of critical residues in TM2, which emerge from the present analysis, includes the aforementioned His83 in Kcv (Fig. 7).

### The critical amino acid positions are also relevant in the pore modules from complex channels

Next, we analyzed the equivalent pore modules from more complex channels (Fig. 1). The latter include the pores of KvATP, Eag1, HCN1, and hERG from which the pore module was cut out in silico. Additionally, also the pore module from the bacterial Kv channel KvLm-PM (Table 1) was included. This is an interesting case, because this pore was experimentally separated from the voltage sensor domain before crystallization (Santos et al. 2012). The same pore module is functional in the context of the whole protein but also as an isolated pore.

The analysis was further supplemented by a representative of a K2P channel and two TRPV channels. These channels were included into the analysis, because the pore of K2P channels corresponds structurally to the alpha subunits of two joined pores from the aforementioned $\hbox {K}^+$ channels (Fig. 1biv,c). The pores of TRPV channels are interesting for the present analysis, because their global architecture is similar to that of the canonical $\hbox {K}^+$ channels. However, in spite of this similarity, TRPV channels exhibit very different ion selectivity and gating features from canonical $\hbox {K}^+$ channels (Huynh et al. 2016).

The results of the analyses show that the critical amino acids, which correspond to 71 and 76 in KcsA are also detected with a high scoring value in all the pore modules of $\hbox {K}^+$ channels with a 6 TMD type architecture (Fig. 1bii). Like in KcsA and the other Kir-type channels, all the pores from Kv channels also exhibit small clusters of important amino acids exclusively in the inner transmembrane domain. This finding is consistent with the general view that this domain has a central importance in the gating of $\hbox {K}^+$ channels (Magidovich and Yifach 2004). The precise localization of the critical residues, however, seems more variable among the different channels than those in the filter domain. One reason for this variability could be the difference in the length of the loop, which connects the filter with the downstream transmembrane domain. To account for this variability, we aligned the data to the start of the transmembrane domain of each of the Kv channels. The plot in Fig. 7 shows that the clusters of critical amino acids in the transmembrane domain occur after this correction in similar positions. This underscores a conserved mechanical connectivity in these channels independent on whether they are ligand or voltage gated. An interesting observation is that the critical residues in the respective transmembrane domain are not identical among very similar channels like Eag1, hERG, and HCN1. This discrepancy may originate from the fact that the structure of hERG was solved in the open state, while those of the two others are from a closed channel (Wang and MacKinnon 2017). Comparison of the respective structures shows that large deviations between an open and closed channel occur in particular in the inner transmembrane domain. The location of the critical residues in the inner transmembrane domain may capture this difference in structure.

The data furthermore imply that the pore module maintains its basic mechanical connectivity independent on whether it is isolated in Kir-type channels or connected to a voltage-sensing domain in Kv channels. This finding is in good agreement with experimental data on the KvLm channel, in which the pore is functional in the entire protein as well as in an isolated form (Santos et al. 2012).

The data further suggest that differences in ion selectivity are not affected by this mechanical properties of the pore; there is no apparent difference between all the $\hbox {K}^+$ selective channels and channels like NaK and HCN1, which also exhibit a conductance for Na$^+$ (Lee et al. 2005; Alam and Jiang 2009b). Furthermore, the conserved mechanical connectivity in the pore module seems not to be responsible for the difference in voltage sensitivity; there is no obvious difference between the outward rectifying Eag1 (Whicher and MacKinnon 2016) and the inward rectifying HCN1 channel (Lee et al. 2005).

In recent studies, we used anisotropic network models of HCN1 to understand the mechanical connections, which are involved in the modulation of gating of this channel by cAMP binding in the cytosolic termini (Gross et al. 2018; Porro et al. 2019). In the context of the present results, we asked the question whether the critical residues, which are highlighted in the reduced model (Fig. 7), maintain their importance in the full protein. To answer this question, we performed an analysis on the full channel structure and identified the same residues as in the isolated pore (Table 1). This again suggests the existence of crucial interactions between the filter and the inner transmembrane domain. Their relative importance seems to be maintained even in the full channel. Furthermore, these findings highlight the stability of our method and its ability to detect important residues even in large channels.

### The pores of KP2 and TRPV channels differ from $\hbox {K}^+$ channels with tetramer architecture

The results from all the canonical $\hbox {K}^+$ channel pores differ from those obtained with the KP2 channel and the TRPVs. The two critical positions in the filter corresponding to 71 and 76 in KcsA are also highlighted in the K2P channel but with a low propensity. The remaining results bear little similarity between the K2P channel and the other $\hbox {K}^+$ channels. This may reflect the fact that K2P channels are unlike the other $\hbox {K}^+$ channels not fourfold symmetric tetramers. Also, unlike the other $\hbox {K}^+$ channels, mammalian K2P channels like the present K2P4.1 contain a large extracellular domain, the cap structure, which extends from the first pore loop (Lolicato et al. 2014). All these structural differences seem to generate a different mechanical connectivity in the pore of these channels.

Scrutiny of the data from the TRPV channels shows that they differ, in spite of their overall similarity in structure (Huynh et al. 2016), dramatically from each other (see Fig. 10). Based on this diversity, which may reflect their difference in gating (Huynh et al. 2016), it is impossible to discuss any similarities and differences from $\hbox {K}^+$ channels.

### Detection of mechanical coupling/uncoupling in channel pores

Using the AIC function as a score value, we identified the amino acids, which are most important for the dynamics of a channel protein. In the next step, we intended to uncover the amino acids which exhibit the strongest and weakest mechanical coupling in the five Kir-type channel proteins, respectively. To obtain this information, we substituted in the simulated annealing procedure the AIC function as a score value with Eqs. (13) and (14) as a measure for the coupling strength. In this procedure, the sum of the square root (Eq.  (13)) was used as a score to estimate the maximal connectivity and the sum of the square (Eq. (14)) as score for the most independent residues.^5^ Since the analysis in Fig. 3 suggested that 4 residues per monomer are sufficient for describing the basic dynamics in the channels, we limited the analysis of connected and disconnected residues also to 4 per monomer.

Figure 4b shows the estimates of the maximal connectivity in the KcsA channel. The critical residues and the frequency of detection are basically the same as those obtained from the AIC function. The data suggest that the importance of the critical amino acids for the function in the KcsA channel originates from their mutual interaction in the mechanical network of the channel protein. The most prominent interactions are located in the filter/pore helix; connectivity in the inner TM domain is also important but apparently less relevant. The general conclusion from this analysis, which suggests a mechanical coupling between these residues in the KcvA channel, is supported by the aforementioned experimental data. Most of the reports on the importance of the critical residues stress a mutual interaction between them in channel gating (Shrivastava and Bahar 2006; Shen et al. 2002; Imai et al. 2010).

**Fig. 8:**
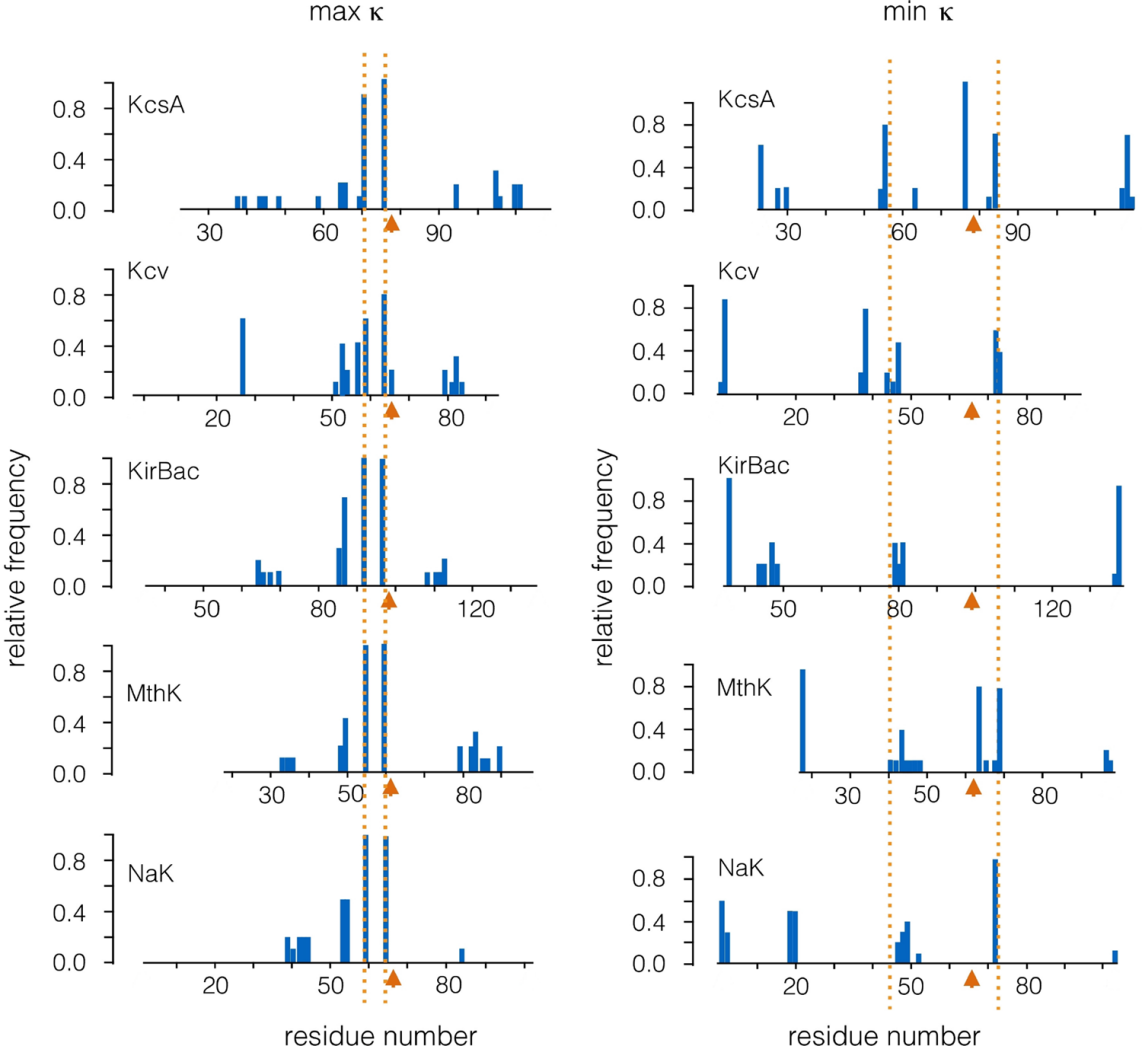
Relative distribution of critical residues in pores of different channel structures with Kir-type architecture. The plot shows the relative frequencies of residues with the 10 highest score values for maximal coupling (left column) and minimal coupling (right column). The plots are aligned to the GXG sequence in the selectivity filter (orange arrow). The dotted lines highlight positions of critical residues, which are observed in all structures

**Fig. 9:**
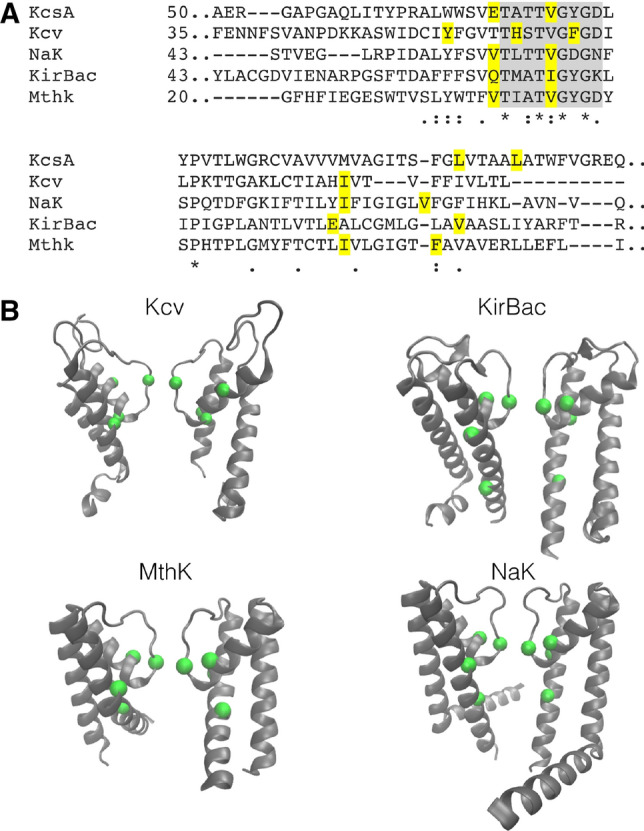
Supplement. The critical four residues determined from the minimal AIC value in the KcsA structure are also detected in all four other Kir-type channels. **a** Multiple sequence alignment of the four channel proteins. The selectivity filter sequence, which is conserved in the $\hbox {K}^+$ selective channels and deviates in a critical amino acid in NaK, is highlighted in gray. The residues with the maximal score in the AIC value in each channel are highlighted in yellow. **b** Localization of the critical residues (green spheres) in two of the four monomers from each channel

**Fig. 10:**
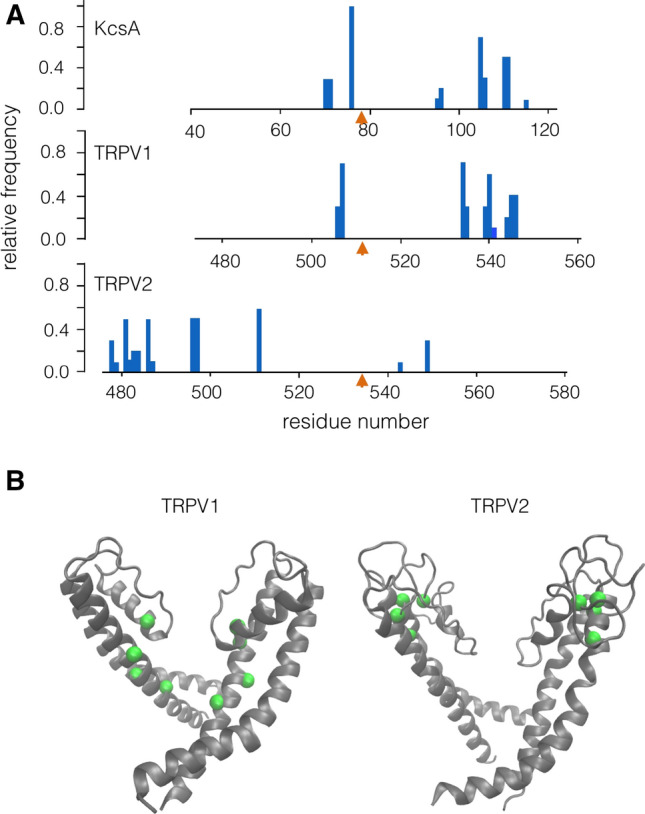
Supplement. Relative distribution of critical residues in pores of two TRPV channels. Analysis as in Fig. 4 for TRPV1 and TRPV2 proteins (**a**). The plot shows the relative frequencies of residues with the 10 best AIC. The data from KcsA (Fig. 4b) are shown in top row for reference. The plots are aligned to the GXG sequence in the selectivity filter (orange arrow). **b** Localization of the critical residues (green spheres) in two of the four monomers from each channel

The general implication of the discovered couplings for the function of all channel pores is again supported by a comparison of the results from all channels. For all the five Kir-type pores, the top scores for connectivity are found for the residues in the pore helix and the filter, which correspond to E71 and V76 in KcsA (Fig. 8left panel). Other critical residues occur in the inner transmembrane domain but at different distances from the selectivity filter. Interesting to note is that, in all channels, an additional more or less pronounced peak of residues occurs just upstream of the pore helix. In KcsA, this peak is located around residues R64,A65.

The analysis of the most disconnected residues in the KcsA channel (Fig. 4c) provides an additional test for consistency of the method. This analysis should only pick up noise in the system from the most flexible parts of the ion channel, namely loops and the termini of the protein. With this procedure, we, indeed, uncovered two residues in clusters around P55 and V84 and two at the end of each transmembrane domain (A23, E118). The inherent high degree of flexibility of the ends of transmembrane domains bears the hazard that the latter positions are an artifact of a truncation of the pores. To test this assumption, we repeated the analysis with the elongated version of the KcsA structure (Fig. 5c,d). In this structure, we found that one of the disconnected residues was again identified at the end of the elongated helix domain. This implies that the ends of the protein structures with the high mobility are maximally disconnected. This is relevant for a protein like the Kcv channel in which the total structure is identical to that analyzed here. In the case of truncated structures like those of KirBac and KcsA, the identification of independent residues in these positions is presumably artifacts and not further considered here.

The remaining amino acids, with a high degree of independency in the KcsA channel (Fig. 4c) and in all other Kir-type channels (Fig. 8), are as expected associated with the flexible loops, which connect the outer transmembrane domain with the pore helix (=turret domain) and with the loop, which connects the selectivity filter with the inner TM domain. We anticipate that this information about less critical regions of a protein is helpful in protein design, e.g., for the question of where to attach an additional domain without disrupting the structure.

## Conclusions

The present study shows that anisotropic network models of channel pores with a similar architecture can be further simplified to four critical residues, which represent the basic mechanical interactions in these proteins. It is striking to find that this analysis identifies with high precision in all cases a pair of residues in the pore helix and the selectivity filter, which are well known for their mechanical interaction and functional importance. The analysis further highlights small regions in the inner TM domains, which are also known for their contribution to channel gating. Important to note is that the same or similar positional hot spots are detected in all the channel pores, even though they differ in their ion selectivity and gating. Also some of them represent the entire monomer of a channel protein, while others are only truncated versions of the full-length channel monomer. This means that the critical residues, which were identified here, must be important for the most basic functional property of these proteins including ion conductance and stochastic switching between an open and closed state. We anticipate that this information on an architectural principle in a channel protein could be valuable for the design of synthetic channel pores.

With the above-described objective functions to obtain mappings $\varvec{\mu }$, we were able to (a) perform model selection based on the AIC, (b) identify strongly coupled, and (c) weakly coupled residues. (b) and (c) are achieved by the choice of $p=2$ and $p=1/2$ in the respective $l_p$ norm above (and the implied curvature). While we focus here on structural-functional building principles of ion channels [and (a)–(c) are sufficient to this end], it seems worthwhile to use our method employing other loss functions to “assign” coupling/representation scores to individual residues. We hope to pursue this in a future study.

While previous, important studies based on atomistic molecular dynamics simulations (Pan et al. 2011; Li et al. 2018; Conti et al. 2016) were able to obtain detailed, (quasi-)quantitative insight, our method gives qualitative insight in a completely other dimension: MD is a sophisticated method to understand concrete atomistic systems; our approach solely tries to identify mechano-structural building schemes via identifying important residues in this regard. While, in principle, MD would be capable to achieve this, one would need to run thousands of independent MD simulations for one channel alone. In answering design questions—e.g., how to fuse independently evolved channel modules—the underlying combinatorial optimization problem is even more demanding on the number of dynamical simulations and thus MD, while with desirable accuracy, seems to be prohibitively expensive for this role. The fast computational runtime of our method allows for much greater combinatorial “scans” of important residues and also for the analysis of whole structural databases. Furthermore, our procedure is well suited to be used in combination with other advanced procedures built upon ANMs like, e.g., contact switch-offs within the linear response theory (Gross et al. 2018).

As we eventually identify residues under high selection pressure (they contribute to the mechano-functional fitness of the molecular phenotype), in future work, one may try to combine coevolutionary methods (Morcos et al. 2011; Schmidt and Hamacher 2017) to gain evolutionary insights into proteins with the method laid out above. For simple enzymes, a somewhat similar approach to combine mechanical dynamics and evolutionary signatures can be found in (Hamacher 2008).

### Electronic supplementary material

Below is the link to the electronic supplementary material.

Supplementary file1 (PDF 2362 kb)

## Figures and Tables

**Fig. 11:**
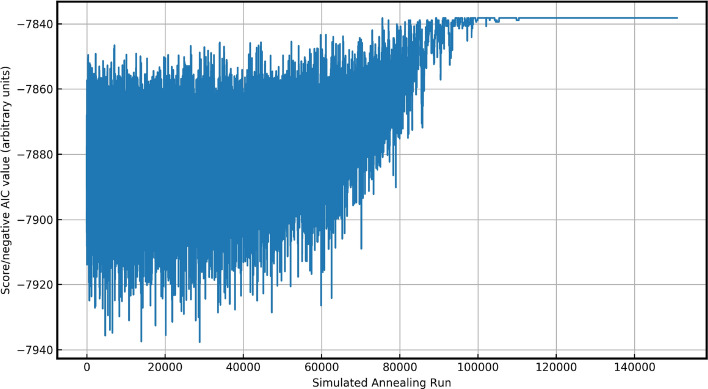
Supplement. Convergence of scores for an execution of Algorithm 1 for AIC minimization of KcsA (1BL8) with a total number of $|{\mathcal{M}}|=16$ model residues. The scores are calculated by Eq. (12) with $k = |{\mathcal{M}}|(|{\mathcal{M}}|-1)/2 = 120$ optimization parameters, as we optimize in every pairwise coupling between the model residues

## Appendix

### Consistency of $D_{KL}$ minimization and configuration integral

Apart from gauge transformations, the minimization of Eq. (2) w.r.t. model potential *U* is equivalent to solving the configuration integral in Eq. (1). To show this, Eq. (1) can be rewritten as:

15 $$\begin{aligned} q\left( {{\bf r}}_m\right) = \int \mathrm {d} {{\bf r}}_t ~ \delta \left( \varvec{\mu }\left( {{\bf r}}_t\right) - {{\bf r}}_m \right) ~ p\left( {{\bf r}}_t\right) \end{aligned}$$

with

16 $$\begin{aligned} q\left( {{\bf r}} \right)&:= \frac{\mathrm {exp}\left[ -\beta U( {{\bf r}}) \right] }{Z_q}, \end{aligned}$$

17 $$\begin{aligned} p({{\bf r}})&:= \frac{\mathrm {exp}\left[ -\beta V\left( {{\bf r}}\right) \right] }{Z_p} \end{aligned}$$

and $Z_q$, $Z_p$ being the corresponding partition functions. Eq. (15) is, apart from gauge transformations $U\rightarrow U+\mathrm {const}$, unambigously related to Eq. (1).

On the other hand, the derivative of the Kullback–Leibler divergence in Eq. (2) is:

18 $$\begin{aligned} \frac{\partial D_{KL}}{\partial U\left( {{\bf r}}_m\right) }= & {} - \frac{\partial }{\partial U({{\bf r}}_m)} \int \mathrm {d}{{\bf r}}_t ~ p\left( {{\bf r}}_t\right) ~ \mathrm {ln}\left[ q\left( \varvec{\mu }({{\bf r}}_t)\right) \right] \end{aligned}$$

19 $$\begin{aligned}= & {} - \int \mathrm {d}{{\bf r}}_t ~ \frac{p\left( {{\bf r}}_t\right) }{q\left( \varvec{\mu }\left( \mathrm{\bf r}_t\right) \right) } \frac{\partial }{\partial U({{\bf r}}_m)} \left\{ \frac{\mathrm {exp}\left[ -\beta U\left( \varvec{\mu }\left( {{\bf r}}_t\right) \right) \right] }{Z_q} \right\} \end{aligned}$$

20 $$\begin{aligned}= & {} \int \mathrm {d}{{\bf r}}_t ~ \frac{p\left( {{\bf r}}_t\right) }{q\left( \varvec{\mu }\left( {{\bf r}}_t\right) \right) } \cdot \left\{ \frac{\mathrm {exp}\left[ -\beta U\left( \varvec{\mu }\left( {{\bf r}}_t\right) \right) \right] }{Z_q^2} \cdot \frac{\partial }{\partial U\left( {{\bf r}}_m\right) }\right. \nonumber \\&\int \mathrm {d}\mathrm{\bf s} ~ \mathrm {exp}\left[ -\beta U(\mathrm{\bf s}) \right] \nonumber \\&\left. + \frac{\mathrm {exp}\left[ -\beta U\left( \varvec{\mu }\left( {{\bf r}}_t\right) \right) \right] }{Z_q} \cdot \beta \delta \left( \varvec{\mu }({{\bf r}}_t) - {{\bf r}}_m \right) \right\} \end{aligned}$$

21 $$\begin{aligned}= & {} \int \mathrm {d}{{\bf r}}_t ~ \frac{p\left( {{\bf r}}_t\right) }{q\left( \varvec{\mu }\left( {{\bf r}}_t\right) \right) } \left\{ -\beta ~ q(\varvec{\mu }({{\bf r}}_t)) ~ q\left( {{\bf r}}_m\right) \right. \nonumber \\&\left. + \beta ~ q\left( \varvec{\mu }\left( {{\bf r}}_t\right) \right) ~ \delta \left( \varvec{\mu }({{\bf r}}_t) - {{\bf r}}_m \right) \right\} \end{aligned}$$

22 $$\begin{aligned}= &\beta \cdot \left\{ -q\left( {{\bf r}}_m\right) ~ \int \mathrm {d}{{\bf r}}_t ~ p\left( {{\bf r}}_t\right) + \int \mathrm {d}{{\bf r}}_t ~ p\left( {{\bf r}}_t\right) \delta \left( \varvec{\mu }\left( {{\bf r}}_t\right) - {{\bf r}}_m \right) \right\} \end{aligned}$$

23 $$\begin{aligned}= &\beta \cdot \left\{ -q\left( {{\bf r}}_m\right) + \int \mathrm {d}{{\bf r}}_t ~ p\left( {{\bf r}}_t\right) \delta \left( \varvec{\mu }\left( {{\bf r}}_t\right) - {{\bf r}}_m \right) \right\} . \end{aligned}$$

For an extremal point, $\frac{\partial D_{KL}}{\partial U({{\bf r}}_m)} = 0$ holds, and we thus directly obtain Eq. (15). $\quad \Box$

### Structural analysis of Kir-type channels

Figure 9 shows the localization of the critical residues in the alignment (A) and the pdb structures (B) of the Kir-type channels.

### Analysis of TRPV channels

Figure 10 shows the identified critical residues of two TRPV channels.

### Settings and analysis of the simulated annealing

We use a total number of $N_\mathrm {total} \ge 10^5$ iterations for the simulated annealing routine. The value for the initial temperature $T_\mathrm {start}$ (final temperature $T_\mathrm {end}$) is chosen, such that an acceptance probability of $p_\mathrm {accept}^\mathrm {start} \ge 0.99$ ($p_\mathrm {accept}^\mathrm {end} \le 0.01$) should be achieved. This is done by a heuristic pre-calculation via bisection: we start with a random mapping and perform a Metropolis sampling according to Algorithm 1, but with a fixed temperature. We determine the corresponding acceptance ratios. Subsequently, we adjust the temperature to come closer to the desired value and perform the next sampling. These steps are repeated until convergence. The cooling factor is chosen as $\alpha = 0.99$ which, together with the above parameters, determines the value of $N_\mathrm {sub}$ (number of samplings with a constant temperature).

We perform ten independent runs of Algorithm 1, each having a different seed for the random number generation. We then concatenate the scores from all runs and choose the mapping according to the highest score. Figure 11 shows the convergence of scores of a single exemplary run for the AIC minimization of KcsA (1BL8). In this case, the mapping with the highest score is identical in every one of the ten independent runs.

### AIC values of all channels with a Kir-type architecture

Table 2 shows the optimized AIC values of the Kir-type channels for different numbers of model residues.

**Table 2:** AIC values (arbitrary units) of all considered ion channels with a Kir-type architecture for different numbers of model residues

Channel	pdb code	AIC (3 model res. per monomer)	AIC (4 model res. per monomer)	AIC (5 model res. per monomer)
KcsA (Doyle et al. 1998)	1BL8	7858	7838	7850
KcsA (Uysal et al. 2009)	3EFF	11344	11327	11342
KcsA (Uysal et al. 2011)	3PJS	11338	11324	11338
Kcv (Tayefeh et al. 2009)	-	7630	7618	7640
KirBac 3.1 (Clarke et al. 2010)	2WLJ	8359	8342	8356
MthK (Posson et al. 2013)	4HYO	6613	6597	6611
NaK (Shi et al. 2006)	2AHY	8361	8343	8358

## Footnotes

1 In the following, we focus on the AIC as it allows for an analytical treatment due to the Gaussian likelihood (as described in Sect. 2). While the application of other metrics like the Bayesian information criterion (Konishi and Kitagawa (2007)) is also feasible, it needs to be based on a real sample of data points (here: configurations). While this is possible in principle to create, such a numerical approach seems to rather expansive in compute time without much gain.

2 In the following, we will use both terms interchangeably, i.e., we will either *minimize the AIC value* or *maximize* $f^\mathrm {sc}$.

3 Our implementation in julia can be found at http://biosrv0.compbiol.bio.tu-darmstadt.de/DKL/.

4 K2P4.1 is a homodimer with length $n=2l$. We accordingly chose our model residues symmetric in both monomers, i.e. $i\in \mathcal {M} \Rightarrow i+l \in \mathcal {M}$.

5 We also performed a stability analysis of these equations by neglecting the outer square [Eq. (13)] and outer root [Eq. (14)] in the simulated annealing optimization. We found all results to be consistent but for only one residue in the KcsA structure 3EFF (His124 instead of Arg121 in Fig. 5c) and slight differences in the decoupled histogram of KirBac 3.1 (Fig. 8, right column).
