# Supplementary material for: Inferring functional units in ion channel pores via relative entropy
Source: Eur Biophys J. 2021 Feb 1;50(1):37–57. doi: 10.1007/s00249-020-01480-7 (PMC7872957; doi:10.1007/s00249-020-01480-7)
Supplement: Supplementary file 1 — Supplementary file1 (PDF 2362 kb) [file 249_2020_1480_MOESM1_ESM.pdf]

European Biophysics Journal manuscript No.
(will be inserted by the editor)

Inferring functional units in ion channel pores via relative entropy

Michael Schmidt · Indra Schroeder · Daniel Bauer · Gerhard Thiel · Kay Hamacher

Received: date / Accepted: date

1 Appendix

1.1 Consistency of D_{KL} minimization and configuration integral

Apart from gauge transformations, the minimization of Eq. (2, main manuscript) w.r.t. model potential U is equivalent to solving the configuration integral in Eq. (1, main manuscript). To show this, Eq. (1, main manuscript) can be rewritten as

$$q(\mathbf{r}_m) = \int \mathrm{d}\mathbf{r}_t \; \delta\left(\boldsymbol{\mu}(\mathbf{r}_t) - \mathbf{r}_m\right) \; p(\mathbf{r}_t) \tag{1}$$

with

$$q(\mathbf{r}) := \frac{\exp\left[-\beta U(\mathbf{r})\right]}{Z_q}, \tag{2}$$

$$p(\mathbf{r}) := \frac{\exp\left[-\beta V(\mathbf{r})\right]}{Z_p} \tag{3}$$

M. Schmidt
Department of Physics, TU Darmstadt, Karolinenpl. 5, 64289 Darmstadt, Germany
E-mail: schmidt@cbs.tu-darmstadt.de

I. Schroeder
Department of Biology, TU Darmstadt, Schnittspahnstr. 10, 64287 Darmstadt, Germany

D. Bauer
Department of Biology, TU Darmstadt, Schnittspahnstr. 10, 64287 Darmstadt, Germany

G. Thiel
Department of Biology, TU Darmstadt, Schnittspahnstr. 10, 64287 Darmstadt, Germany
E-mail: thiel@bio.tu-darmstadt.de

K. Hamacher
Department of Physics, Department of Biology, Department of Computer Science, TU Darmstadt, Schnittspahnstr. 10, 64287 Darmstadt, Germany
E-mail: hamacher@bio.tu-darmstadt.de

and Z_q, Z_p being the corresponding partition functions. Eq. (1) is, apart from gauge transformations $U \to U + \text{const}$, unambigously related to Eq. (1, main manuscript).

On the other hand, the derivative of the Kullback-Leibler divergence in Eq. (2, main manuscript) is

$$\frac{\partial D_{KL}}{\partial U(\mathbf{r}_m)} = -\frac{\partial}{\partial U(\mathbf{r}_m)} \int \mathrm{d}\mathbf{r}_t \; p(\mathbf{r}_t) \; \ln\left[q(\boldsymbol{\mu}(\mathbf{r}_t))\right] \tag{4}$$

$$= -\int \mathrm{d}\mathbf{r}_t \; \frac{p(\mathbf{r}_t)}{q(\boldsymbol{\mu}(\mathbf{r}_t))} \frac{\partial}{\partial U(\mathbf{r}_m)} \left\{ \frac{\exp\left[-\beta U(\boldsymbol{\mu}(\mathbf{r}_t))\right]}{Z_q} \right\} \tag{5}$$

$$= \int \mathrm{d}\mathbf{r}_t \; \frac{p(\mathbf{r}_t)}{q(\boldsymbol{\mu}(\mathbf{r}_t))} \cdot \left\{ \frac{\exp\left[-\beta U(\boldsymbol{\mu}(\mathbf{r}_t))\right]}{Z_q^2} \cdot \frac{\partial}{\partial U(\mathbf{r}_m)} \int \mathrm{d}\mathbf{s} \; \exp\left[-\beta U(\mathbf{s})\right] \right.$$
$$\left. + \frac{\exp\left[-\beta U(\boldsymbol{\mu}(\mathbf{r}_t))\right]}{Z_q} \cdot \beta \delta\left(\boldsymbol{\mu}(\mathbf{r}_t) - \mathbf{r}_m\right) \right\} \tag{6}$$

$$= \int \mathrm{d}\mathbf{r}_t \; \frac{p(\mathbf{r}_t)}{q(\boldsymbol{\mu}(\mathbf{r}_t))} \left\{ -\beta \; q(\boldsymbol{\mu}(\mathbf{r}_t)) \; q(\mathbf{r}_m) \right.$$
$$\left. + \beta \; q(\boldsymbol{\mu}(\mathbf{r}_t)) \; \delta\left(\boldsymbol{\mu}(\mathbf{r}_t) - \mathbf{r}_m\right) \right\} \tag{7}$$

$$= \beta \cdot \left\{ -q(\mathbf{r}_m) \int \mathrm{d}\mathbf{r}_t \; p(\mathbf{r}_t) + \int \mathrm{d}\mathbf{r}_t \; p(\mathbf{r}_t) \delta\left(\boldsymbol{\mu}(\mathbf{r}_t) - \mathbf{r}_m\right) \right\} \tag{8}$$

$$= \beta \cdot \left\{ -q(\mathbf{r}_m) + \int \mathrm{d}\mathbf{r}_t \; p(\mathbf{r}_t) \delta\left(\boldsymbol{\mu}(\mathbf{r}_t) - \mathbf{r}_m\right) \right\}. \tag{9}$$

For an extremal point $\frac{\partial D_{KL}}{\partial U(\mathbf{r}_m)} = 0$ holds and we thus directly obtain Eq. (1). □

1.2 Structural analysis of Kir type channels

Figure 1 shows the localization of the critical residues in the alignment (A) and the pdb structures (B) of the Kir type channels.

1.3 Analysis of TRPV channels

Figure 2 shows the identified critical residues of two TRPV channels.

1.4 Settings and analysis of the simulated annealing

We use a total number of $N_{\text{total}} \geq 10^5$ iterations for the simulated annealig routine. The value for the initial temperature T_{start} (final temperature T_{end})

A

```
KcsA      50..AER---GAPGAQLITYPRALWWSVETATTVGYGDL
Kcv       35..FENNFSVANPDKKASWIDCIYFGVTTHSTVGFGDI
NaK       43..------STVEG---LRPIDALYFSVVTLTTVGDGNF
KirBac    43..YLACGDVIENARPGSFTDAFFFSVQTMATIGYGKL
Mthk      20..-------GFHFIEGESWTVSLYWTFVTIATVGYGDY
                                  .::: . * :*:* *.

KcsA        YPVTLWGRCVAVVVMVAGITS-FGLVTAALATWFVGREQ..
Kcv         LPKTTGAKLCTIAHIVT---V-FFIVLTL----------
NaK         SPQTDFGKIFTILYIFIGIGLVFGFIHKL-AVN-V---Q..
KirBac      IPIGPLANTLVTLEALCGMLG-LAVAASLIYARFT---R..
Mthk        SPHTPLGMYFTCTLIVLGIGT-FAVAVERLLEFL----I..
             *    .   .    .      : .
```

B

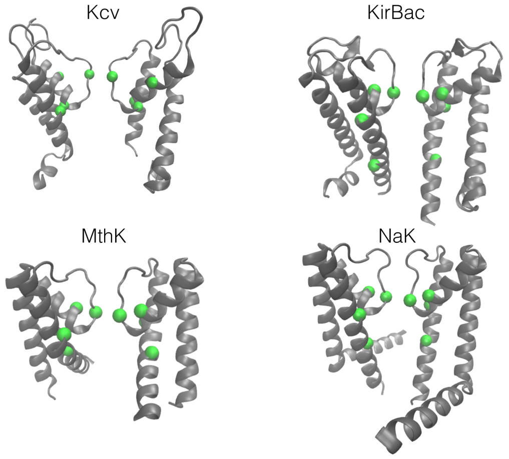

Fig. 1 Supplement. The critical four residues determined from the minimal AIC value in the KcsA structure are also detected in all four other Kir type channels. (A) Multiple sequence alignment of the four channel proteins. The selectivity filter sequence, which is conserved in the K^+ selective channels and deviates in a critical amino acid in NaK is highlighted in grey. The residues with the maximal score in the AIC value in each channel are highlighted in yellow. (B) Localization of the critical residues (green spheres) in two of the four monomers from each channel.

is chosen such that an acceptance probability of $p_{\text{accept}}^{\text{start}} \geq 0.99$ ($p_{\text{accept}}^{\text{end}} \leq 0.01$) should be achieved. This is done by a heuristic pre-calculation via bisection: We start with a random mapping and perform a Metropolis sampling according to Algorithm 1, but with a fixed temperature. We determine the corresponding acceptance ratios. Subsequently, we adjust the temperature to come closer to the desired value and perform the next sampling. These steps are repeated until convergence. The cooling factor is chosen as $\alpha = 0.99$ which, together with the above parameters, determines the value of N_{sub} (number of samplings with a constant temperature).

We perform ten independent runs of Algorithm 1, each having a different seed for the random number generation. We then concatenate the scores from all runs and choose the mapping according to the highest score. Fig. 3 shows the convergence of scores of a single exemplary run for the AIC minimization of KcsA (1BL8). In this case, the mapping with the highest score is identical in every one of the ten independent runs.

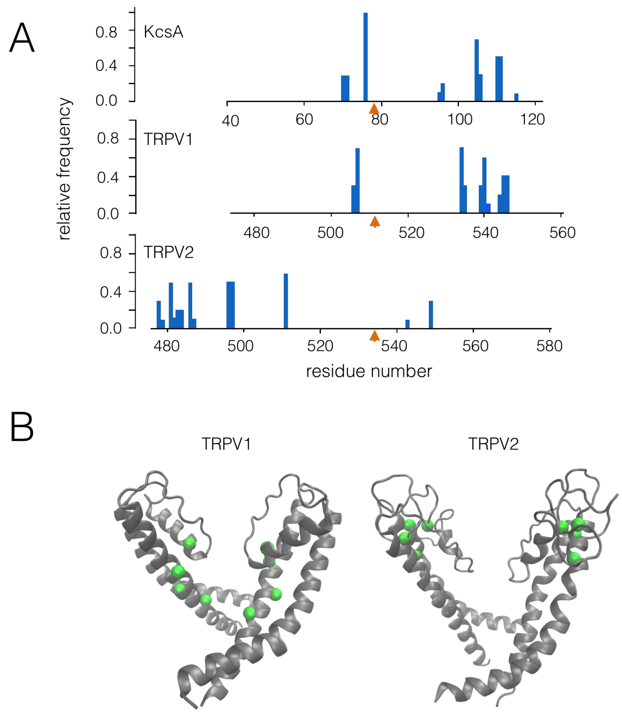

Fig. 2 Supplement. Relative distribution of critical residues in pores of two TRPV channels. Analysis as in Fig. 4 (main manuscript) for TRPV1 and TRPV2 proteins (A). The plot shows the relative frequencies of residues with the 10 best AIC. The data from KcsA (Fig. 4 B, main manuscript) are shown in top row for reference. The plots are aligned to the GXG sequence in the selectivity filter (orange arrow). (B) Localization of the critical residues (green spheres) in two of the four monomers from each channel.

1.5 AIC values of all channels with a Kir type architecture

Table 1 shows the optimized AIC values of the Kir type channels for different numbers of model residues.

References

Clarke OB, Caputo AT, Hill AP, Vandenberg JI, Smith BJ, Gulbis JM (2010) Domain reorientation and rotation of an intracellular assembly regulate conduction in kir potassium channels. Cell 141(6):1018 – 1029, DOI https://doi.org/10.1016/j.cell.2010.05.003, URL http://www.sciencedirect.com/science/article/pii/S0092867410005015

Doyle DA, Cabral JM, Pfuetzner RA, Kuo A, Gulbis JM, Cohen SL, Chait BT, MacKinnon R (1998) The structure of the potassium channel: Molecular basis of k^+ conduction and selec-

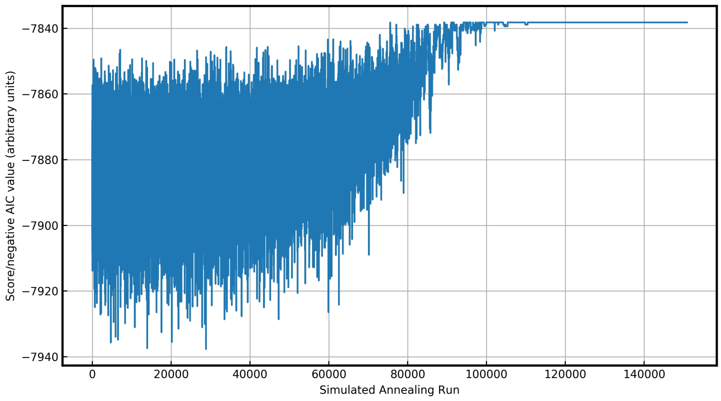

Fig. 3 Supplement. Convergence of scores for an execution of Algorithm 1 for AIC minimization of KcsA (1BL8) with a total number of $|\mathcal{M}| = 16$ model residues. The scores are calculated by Eq. (12, main manuscript) with $k = |\mathcal{M}|(|\mathcal{M}| - 1)/2 = 120$ optimization parameters, as we optimize in every pairwise coupling between the model residues.

Table 1 AIC values (arbitrary units) of all considered ion channels with a Kir type architecture for different numbers of model residues.

Channel	pdb code	AIC (3 model res. per monomer)	AIC (4 model res. per monomer)	AIC (5 model res. per monomer)
KcsA (Doyle et al., 1998)	1BL8	7858	7838	7850
KcsA (Uysal et al., 2009)	3EFF	11344	11327	11342
KcsA (Uysal et al., 2011)	3PJS	11338	11324	11338
Kcv (Tayefeh et al., 2009)	-	7630	7618	7640
KirBac 3.1 (Clarke et al., 2010)	2WLJ	8359	8342	8356
MthK (Posson et al., 2013)	4HYO	6613	6597	6611
NaK (Shi et al., 2006)	2AHY	8361	8343	8358

tivity. Science 280(5360):69–77, DOI 10.1126/science.280.5360.69, URL http://science.sciencemag.org/content/280/5360/69, http://science.sciencemag.org/content/280/5360/69.full.pdf

Posson DJ, McCoy JG, Nimigean CM (2013) The voltage-dependent gate in mthk potassium channels is located at the selectivity filter. Nature Structural & Molecular Biology pp 159–166

Shi N, Ye S, Alam A, Chen L, Jiang Y (2006) Atomic structure of a na+- and k+-conducting channel. Nature 440:570–4, DOI 10.1038/nature04508

Tayefeh S, Kloss T, Kreim M, Gebhardt M, Baumeister D, Hertel B, Richter C, Schwalbe H, Moroni A, Thiel G, Kast SM (2009) Model development for the viral kcv potassium channel. Biophysical Journal 96(2):485 – 498, DOI https://doi.org/10.1016/j.bpj.2008.09.050, URL http://www.sciencedirect.com/science/article/pii/S0006349508000659

Uysal S, Vásquez V, Tereshko V, Esaki K, Fellouse FA, Sidhu SS, Koide S, Perozo E, Kossiakoff A (2009) Crystal structure of full-

length kcsa in its closed conformation. Proceedings of the National Academy of Sciences 106(16):6644–6649, DOI 10.1073/pnas.0810663106, URL https://www.pnas.org/content/106/16/6644, https://www.pnas.org/content/106/16/6644.full.pdf

Uysal S, Cuello LG, Cortes DM, Koide S, Kossiakoff AA, Perozo E (2011) Mechanism of activation gating in the full-length kcsa k+ channel. Proceedings of the National Academy of Sciences 108(29):11896–11899, DOI 10.1073/pnas.1105112108, URL https://www.pnas.org/content/108/29/11896, https://www.pnas.org/content/108/29/11896.full.pdf
